# Aurora kinase A regulates Survivin stability through targeting FBXL7 in gastric cancer drug resistance and prognosis

**DOI:** 10.1038/oncsis.2016.80

**Published:** 2017-02-20

**Authors:** M Kamran, Z-J Long, D Xu, S-S Lv, B Liu, C-L Wang, J Xu, E W-F Lam, Q Liu

**Affiliations:** 1 Institute of Cancer Stem Cell, Cancer Center, Dalian Medical University, Dalian/State Key Laboratory of Oncology in South China, Cancer Center, Sun Yat-sen University, Guangzhou, China; 2 Department of Hematology, The Third Affiliated Hospital; Institute of Hematology, Sun Yat-sen University, Guangzhou, China; 3 State key Laboratory of Oncology in South China, Collaborative Innovation Center for Cancer Medicine/Department of Gastric Surgery, Sun Yat-sen University Cancer Center, Guangzhou, China; 4 Department of Surgery and Cancer, Imperial College London, London, UK

## Abstract

Aurora kinase A (AURKA) has been implicated in the regulation of cell cycle progression, mitosis and a key number of oncogenic signaling pathways in various malignancies. However, little is known about its role in gastric cancer prognosis and genotoxic resistance. Here we found that AURKA was highly overexpressed in gastric cancer and inversely correlated with disease prognosis. Overexpression of AURKA exacerbated gastric cancer drug resistance through upregulating the expression of the anti-apoptotic protein Survivin. Conversely, we demonstrated that AURKA depletion caused a decrease in Survivin protein levels by increasing its ubiquitylation and degradation. Mass spectrometric analysis revealed that upon AURKA depletion, Survivin bound to the FBXL7 E3 ubiquitin ligase, which induced ubiquitin-proteasome degradation of Survivin. In addition, we showed that AURKA regulated FBXL7 both at the levels of transcription and translation. Moreover, proteomic analysis of nuclear AURKA-interacting proteins identified Forkhead box protein P1 (FOXP1). We next showed that AURKA was required for FBXL7 transcription and that AURKA negatively regulated FOXP1-mediated FBXL7 expression. The physiological relevance of the regulation of Survivin by AURKA through the FOXP1–FBXL7 axis was further underscored by the significant positive correlations between AURKA and Survivin expression in gastric cancer patient samples. Moreover, the AURKA depletion or kinase inhibition-induced apoptotic cell death could be reversed by Survivin ectopic overexpression, further supporting that AURKA regulated Survivin to enhance drug resistance. In agreement, inhibition of AURKA synergistically enhanced the cytotoxic effect of DNA-damaging agents in cancer cells by suppressing Survivin expression. Taken together, our data suggest that AURKA restricts Survivin ubiquitylation and degradation in gastric cancer to promote drug resistance and hence the AURKA–Survivin axis can be targeted to promote the efficacy of DNA-damaging agents in gastric cancer.

## Introduction

Gastric cancer is one of the most common cancers with high incidence of disease-related deaths and poor prognosis.^1^ Currently, surgical resection and chemotherapy are the most effective treatments. However, patients with locally advanced disease respond poorly to chemotherapeutic modalities, reflecting an inherent refractive mechanism against drug-induced cell death.^2^ Several previous reports have attempted to explore the molecular markers that drive drug resistance. These proposed markers and signatures, including PI3K/Akt, NFκB, inhibitors of apoptosis (IAPs) and Bcl-2 family proteins, are highly expressed in gastric cancer and associated with resistance to chemotherapy-induced cell death.^3, 4^

Aurora kinases were first identified in *Drosophila* as key players in chromosomal segregation.^5^ Subsequently, orthologues were also discovered in humans and implicated in the control of distinct and unrelated aspects of mitosis. Human Aurora kinase A (AURKA) is essential for centrosome duplication, maturation and separation.^6^ AURKA is a potent oncogene that has the capacity to transform certain cell lines when overexpressed.^7^ Recent evidence demonstrated that AURKA could regulate c-Myc expression through cooperating with hnRNP K.^8^ AURKA overexpression is also a hallmark of many cancers and can enhance chromosomal instability through centrosome amplification. The human *AURKA* gene maps to chromosome region 20q13.2, which is frequently amplified in different malignancies, including gastric cancer. A previous study showed that AURKA overexpression and amplification are involved in differentiated-type gastric carcinogenesis and the development of aneuploidy, suggesting that it might contribute to the initiation and progression of gastric cancer.^9^ AURKA has also been implicated in taxane and microtubule destabilizing drug resistance;^10^ however, its role in gastric cancer, especially in resistance to DNA-damaging therapeutic agents remains undefined. Importantly, a previous study using comparative genomic hybridization array found that AURKA overexpression in high-risk primary gastric cancer tissues is associated with dysregulated expression of DNA damage response genes, which also include Survivin.^11^

Survivin is the smallest member of human IAPs and has two critical but not yet fully elucidated roles in cell proliferation and survival.^12^ First, Survivin is highly expressed in many human malignancies and can restrict programmed cell death by inhibiting the function of executioner caspases and procaspases. Secondly, Survivin is also part of the chromosomal passenger complex and responsible for recruiting chromosomal passenger complex to mitotic chromosome, thus having a crucial role in genome stability. In addition to these widely studied functions, Survivin also has an important but less well studied role in microtubule stabilization.^13^ Survivin is an oncofetal protein with elevated expression in stem and cancer cells, while expressed at low level in normal adult differentiated cells.^13, 14, 15^ Survivin has been reported to be overexpressed in both solid tumors and hematological malignancies and its overexpression linked to drug resistance in leukemia,^16, 17^ breast cancer,^18^ neuroblastoma^19^ and ovarian cancer.^20^ Survivin expression has both positive and negative effects on clinical prognosis depending on its location. Nuclear Survivin has been associated with a better prognosis, whereas cytoplasmic Survivin is associated with in some cancer types poor clinical outcome.^21^ In gastric cancer, the five-year survival rate of patients with positive Survivin expression is significantly lower than Survivin-negative patients.^22^ Survivin protein undergoes post-translational modifications, including acetylation, phosphorylation and ubiquitylation,^23^ and these processes modulate Survivin activity.

Although there is strong evidence that AURKA and Survivin are simultaneously co-overexpressed in various malignancies, including breast^24^ and chronic lymphocytic leukemia,^25^ relatively little is known about their expression, regulation and function in gastric cancer. In this study, we addressed this question and found that AURKA and Survivin cooperated in gastric cancer development and had a decisive role in resistance to DNA-damaging agents and poor cancer prognosis. Moreover, we revealed that AURKA stabilized Survivin protein by suppressing its protein degradation through negatively regulating Forkhead box protein P1 (FOXP1)-mediated FBXL7 expression.

## Results

### AURKA expression correlates with poor prognosis in gastric cancer

**Figure 1:**
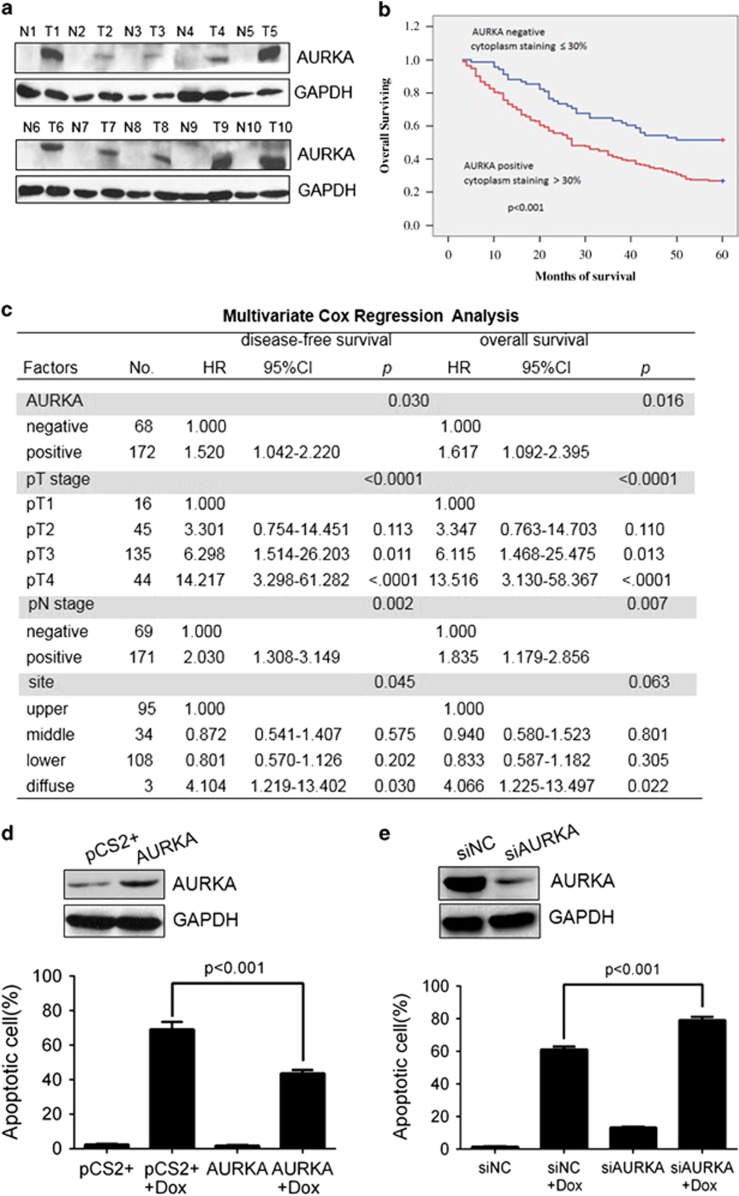
AURKA expression is inversely correlated with gastric cancer prognosis and overrides DNA damage-induced cell death. (**a**) Western blot analysis of AURKA expression in gastric cancer (T) and normal adjacent tissues (N). Whole-tissue extracts were prepared and western blotting analysis performed with the indicated antibodies. GAPDH was used as internal loading control. (**b**) Kaplan–Meier survival analysis of 240 gastric cancer patients. Patients' primary tissues were grouped by high and low AURKA staining values. High AURKA expression was significantly associated with the overall survival (OS) rate. (**c**) Multivariate cox regression analysis of disease-free survivals (DFS) and OS. 95% CI, 95% confidence interval; HR, hazard rate. (**d**) BGC823 cells transfected with cDNA encoding wild-type AURKA were treated with or without doxorubicin (1 μg/ml) for 48 h and subjected to western blot and TUNEL assay. Data represent means±s.e.m. of three independent experiments with significance determined by one-way ANOVA followed by Tukey's multiple comparison test. (**e**) BGC823 cells transfected with AURKA siRNA sequences were treated with or without doxorubicin (1 μg/ml) for 48 h and subjected to western blot and TUNEL assay. Data represent means±s.e.m. of three independent experiments with significance determined by one-way ANOVA followed by Tukey's multiple comparison test.

We first assessed the relationship between AURKA and gastric cancer prognosis by immunohistochemical analysis. To this end, we studied the expression of AURKA protein in a large cohort of 240 gastric cancer patients and found that 172 (71.7%) subjects had high AURKA expression. To further validate our immunohistochemistry results, we performed western blot analysis of paired samples from gastric tumors and their adjacent normal tissues. Consistently, we found a higher AURKA expression in gastric tumor tissues compared with their adjacent normal tissues (Figure 1a). Clinicopathologic correlation analysis showed that AURKA positivity was strongly correlated with a number of gastric cancer clinical features. The AURKA staining was positively correlated with tumor stage (*P*=0.006), clinical stage (*P*=0.002), lymph node metastasis (*P*=0.007) and distant metastasis (*P*<0.0001). We did not find a significant correlation between AURKA overexpression and gender, age, sex, tumor size, tumor site and grading (Supplementary Table 1).

Moreover, gastric cancer patients with high AURKA showed significantly poorer disease free survival (DFS, *P*=0.001) and overall survival (OS, *P*<0.001) compared with low AURKA expressing gastric cancer patients (Figure 1b). Univariate analysis showed that survival time also decreased with tumor size (>4 cm) (DFS, *P*<0.001; OS, *P*<0.001), higher tumor stage (pT) classification (DFS, *P*<0.0001; OS, *P*<0.0001), lymph node metastasis (DFS, *P*<0.0001; OS, *P*< 0.0001) and advanced pTNM stage (DFS, *P*<0.0001; OS, *P*<0.0001). There was no statistically significant correlation between DFS or OS and age, sex or tumor grade (Supplementary Table 2). In multivariate, analysis both lymph node metastasis (DFS, *P*=0.002; OS, *P*=0.007) and tumor stage (DFS, *P*<0.0001; OS, *P*<0.0001) were significant independent prognostic factors for survival (Figure 1c). In addition, multivariate analysis also indicated a significant correlation between AURKA expression and survival (DFS, *P*=0.030; OS, *P*=0.016). Thus, we showed that AURKA level, as an independent prognostic factor, was adversely associated with clinical prognosis, suggesting that the poor chemotherapy response in gastric cancer patients might be related to high AURKA expression (Figure 1c).

Importantly, TUNEL assay showed that overexpression of AURKA reversed cell apoptotic death caused by doxorubicin in gastric cancer BGC823 cells (Figure 1d; Supplementary Figure 1). Conversely, AURKA depletion by siRNA effectively increased sensitivity to doxorubicin (Figure 1e), indicating that AURKA could override DNA damage checkpoint to promote cell survival.

### AURKA regulates Survivin expression in gastric cancer cells

**Figure 2:**
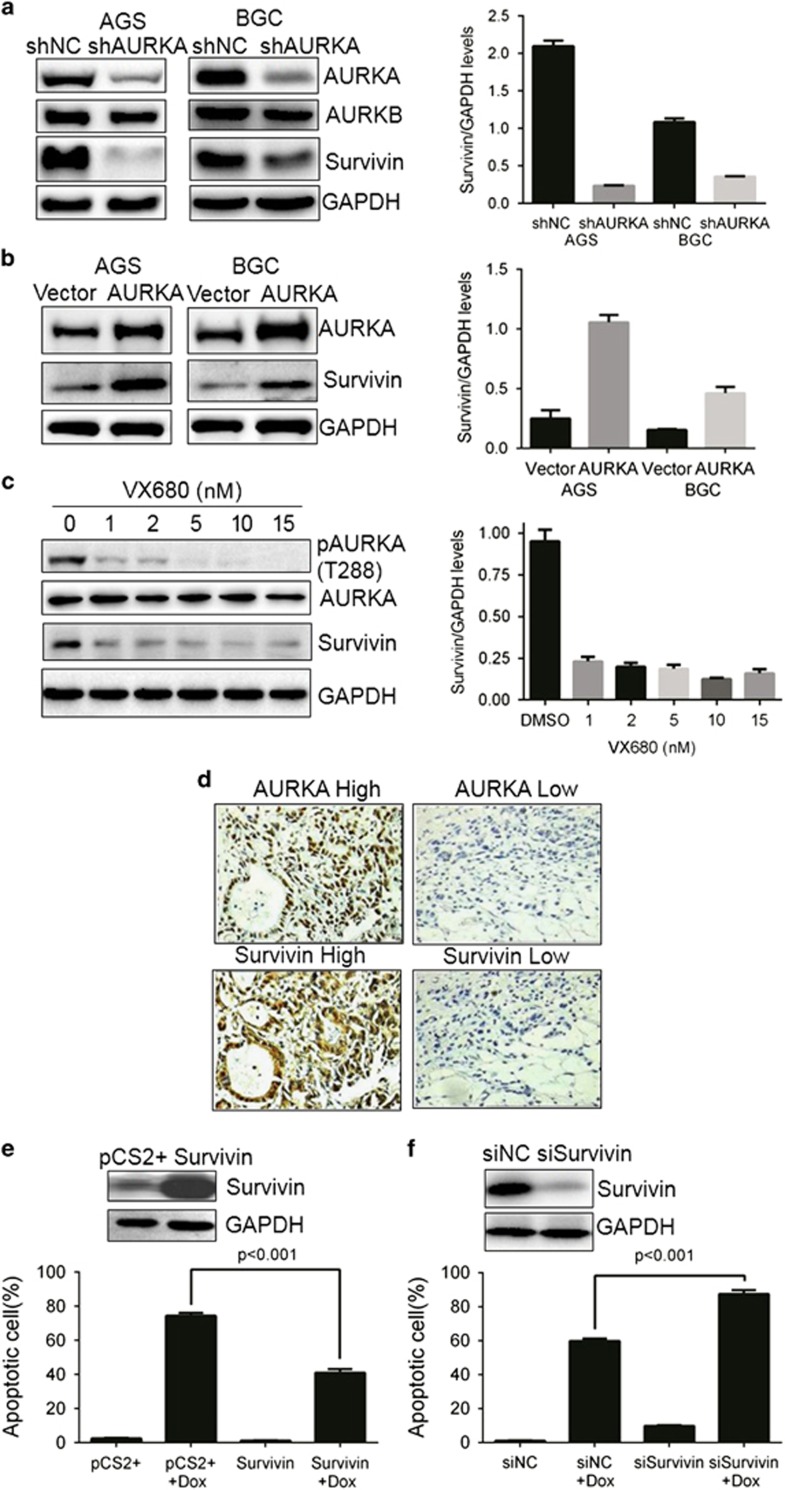
AURKA upregulates Survivin expression and sustains gastric cancer cell survival. (**a**) shRNA-mediated knockdown of AURKA downregulates Survivin protein levels in AGS and BGC823 cells. Cells were infected with lentiviruses expressing shAURKA or control scrambled RNA targeting GFP. A pool of resistant cells was selected by puromycin and cells were cultured in the presence of 1 μg/ml doxycycline. Forty-eight hours after induction, cells were lysed and subjected to western blotting with the indicated antibodies. (**b**) AURKA ectopic expression upregulates Survivin protein levels. Cells were transfected with control and AURKA expressing plasmids. After 24 hours, cells lysates were immunoblotted with the indicated antibodies. (**c**) BGC823 cells were incubated with indicated doses of VX-680 for 24 h before subjected to western blotting with the indicated antibodies. Densitometry was used to quantify the Survivin and GAPDH levels. The relative expression shown (right panel) are means±s.e.m. of the ratios of Survivin to GAPDH. (**d**) Positive correlation between AURKA and Survivin expression in gastric cancer patients. AURKA and Survivin expression was assessed by immunohistochemistry using gastric cancer tissue samples from 62 patients. Representative staining images of one patient with high AURKA and Survivin and one with low expression are shown. Images (magnification 200 ×). Positive correlation between AURKA and Survivin was observed. (**e**) BGC823 cells transfected with cDNA encoding wild-type Survivin were treated with or without doxorubicin (1 μg/ml) for 48 h and subjected to Western blot and TUNEL assay. Data represent means±s.e.m. of three independent experiments with significance determined by one-way ANOVA followed by Tukey's multiple comparison test. (**f**) BGC823 cells transfected with Survivin siRNA sequences were treated with or without doxorubicin (1 μg/ml) for 48 h and subjected to Western blot and TUNEL assay. Data represent means±s.e.m. of three independent experiments with significance determined by one-way ANOVA followed by Tukey's multiple comparison test.

To confirm our hypothesis that AURKA modulates Survivin expression in gastric cancer cells, we depleted endogenous AURKA in gastric cancer cells and examined AURKA and Survivin protein expression. The western blot analysis indicated Survivin expression dramatically decreased in AURKA-depleted AGS and BGC823 gastric cancer cells (Figure 2a), suggesting that AURKA positively regulated Survivin expression. In agreement, AURKA ectopic overexpression in both cells induced Survivin expression levels (Figure 2b), indicating AURKA was an upstream regulator of Survivin expression. It was noteworthy that silencing of AURKA had little or no effects on mRNA levels in AGS and BGC823 cells (Supplementary Figure 2), suggesting that AURKA regulated Survivin at the post-transcriptional level. Further, AURKA small molecule inhibitor VX-680 treatment also resulted in a marked decreased in Survivin protein level in a kinase-dependent manner (Figure 2c), indicating AURKA kinase activity was required for the regulation of Survivin expression. To confirm further that AURKA mediated Survivin upregulation, we performed immunohistochemistry of 62 pairs of gastric cancer specimens and found that AURKA expression was positively correlated with Survivin expression (*r*=0.402; *P*<0.01; Figure 2d; Supplementary Table 3). Considering that co-overexpression of AURKA and Survivin was associated with poor prognosis of gastric carcinomas, we assessed the effect of Survivin expression on drug sensitivity. To achieve this, BGC283 cells were transfected with Survivin cDNA expressing plasmid or siRNA sequences and treated with doxorubicin followed by TUNNEL assay. As shown in Figures 2e and f, elevated Survivin expression was correlated with decreased sensitivity to doxorubicin while Survivin depletion exacerbated cell death compared with control cells in response to the DNA-damaging agent. Together, these data suggested that AURKA regulated Survivin protein expression and upregulation of Survivin levels was the critical mechanism by which AURKA caused gastric cancer cell proliferation and drug resistance to doxorubicin.

### VX-680 synergistically enhances the cytotoxic effect of doxorubicin by suppression of survivin

**Figure 3:**
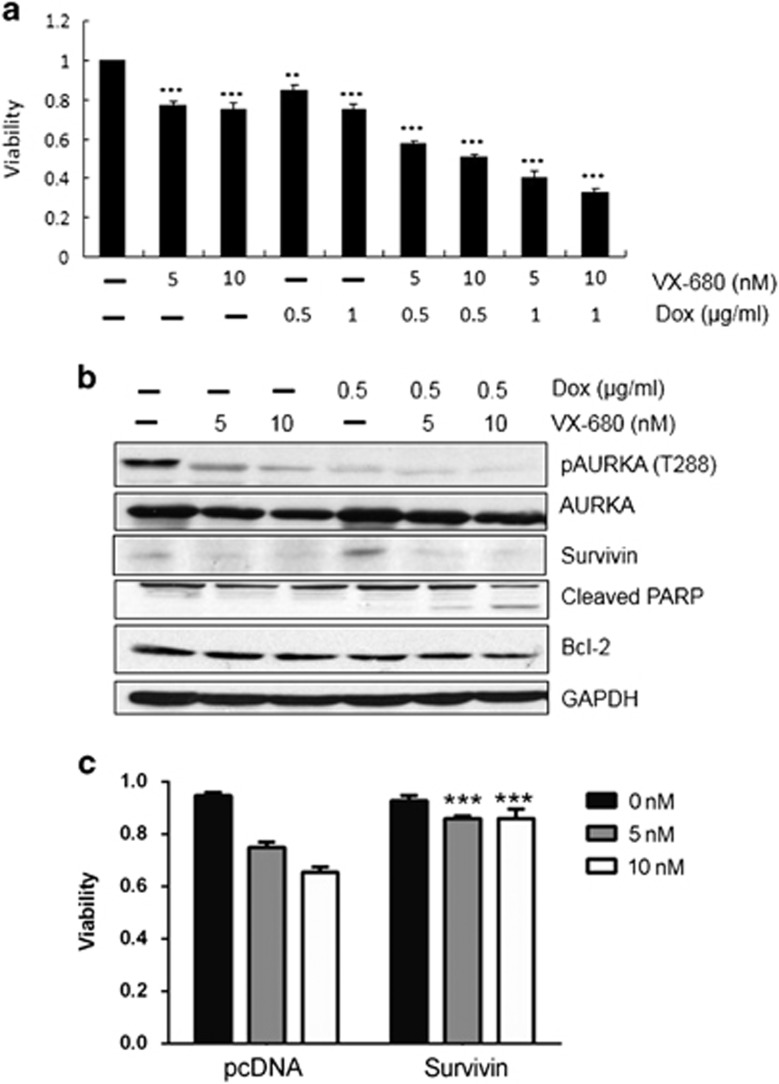
VX-680 synergistically enhances the cytotoxic effect of doxorubicin by suppression of Survivin. (**a**) BGC823 cells were incubated with indicated doses of VX-680, doxorubicin, or combination of both for 24 h before subjected to MTT assay. Column, mean number of survived cells; Bar, s.d.; ***P<0.001 compared with control. (**b**) BGC823 cells were incubated with VX-680, doxorubicin or combination of both for 24 h, and subjected to western blot analysis with AURKA (T288), AURKA, Survivin, Bcl-2, cleaved PARP and GAPDH antibodies. (**c**) Overexpression of Survivin protects gastric cancer cells from VX-680-induced cell death. BGC823 cells transiently overexpressing Survivin were treated with VX-680 for 24 h before subjected to MTT assay. Column, mean number of survived cells; Bar, s.d.; ***very significant: *P*<0.001 compared with control.

We next sought to determine whether the small molecule AURKA kinase inhibitor VX-680 might potentiate the genotoxic effects of doxorubicin. To this end, BGC823 cells were treated with VX-680, doxorubicin, or a combination of both, and the proliferation of the cells analyzed by MTT assay. The combination of these two agents strongly reduced cell proliferation. The inhibition rate of VX-680 and 0.5 μg/ml doxorubicin combination were 42.4% (*q*=1.19) and 49.7% (*q*=1.35), while VX-680 and 1 μg/ml doxorubicin combination were 59.7% (*q*=1.39) and 67.4% (*q*=1.51), respectively (Figure 3a). Western blot showed that combination of these two agents strongly reduced AURKA activity, with an increase in cleaved PARP expression. Most importantly, Survivin expression was induced by doxorubicin but was strongly reduced by the combination treatment (Figure 3b). Moreover, VX-680 induced cell death was significantly (*P*<0.001) rescued by ectopic expression of Survivin (Figure 3c), indicating that Survivin was one of the key molecules targeted by AURKA signaling. Our results provided evidence that AURKA expression correlated with cell response to chemotherapy and inhibition of AURKA might potentiate the efficacy of chemotherapeutic agent, such as doxorubicin for gastric cancer therapy.

### AURKA suppresses Survivin polyubiquitylation and proteasomal degradation

**Figure 4:**
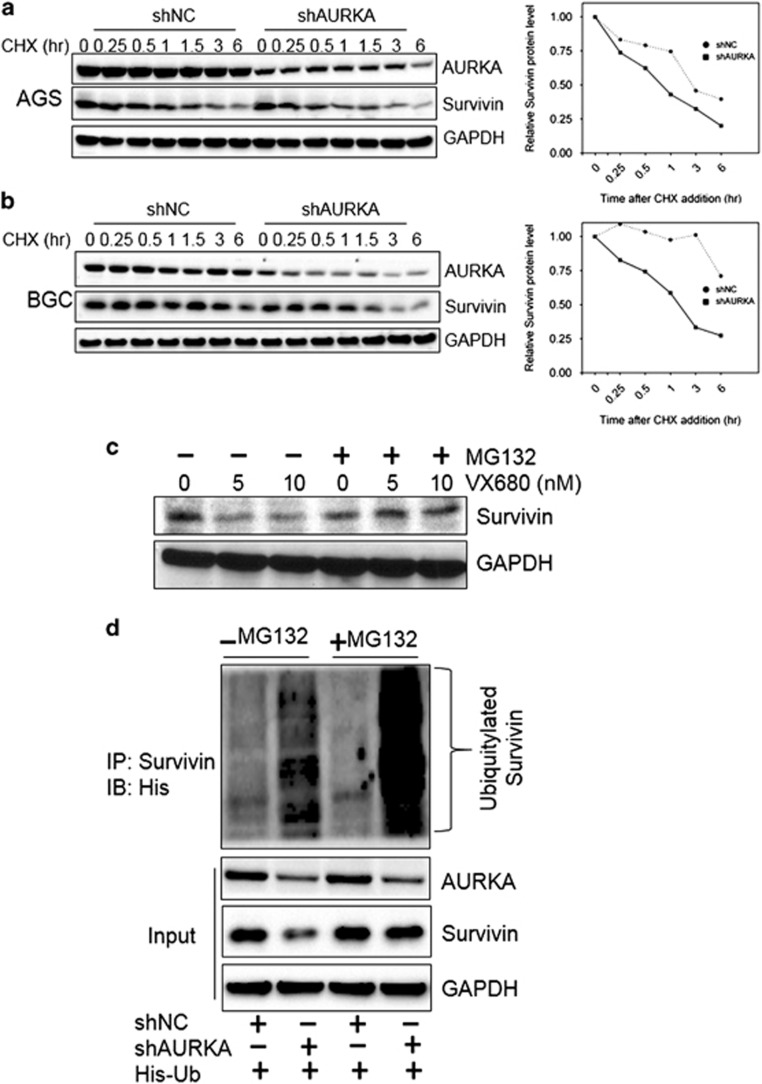
AURKA suppresses the degradation of Survivin in gastric cancer cells. (**a** and **b**) AGS and BGC823 cells stably expressing doxycycline-inducible shAURKA or control cells were cultured in the presence of 1 μg/ml doxycycline. Forty-eight hours after induction, cells treated with 100 μg/ml cycloheximide for indicated times and cell lysates were immunoblotted with the indicated antibodies. Densitometry was used to quantify the Survivin and GAPDH levels. The relative expression shown (right panel) are means±s.d. of the ratios of Survivin to GAPDH. (**c**) BGC823 cells were treated with indicated doses of VX-680, with or without MG132 for 12 h before subjected to western blot analysis with Survivin and GAPDH antibodies. (**d**) BGC823 cells stably expressing doxycycline-inducible shAURKA or control cells were transfected with His-ubiquitin plasmid and cultured in the presence of 1 μg/ml doxycycline. Twenty-four hours after induction, cells were treated with 10 μm MG132 for 6 h and subjected to immunoprecipitation with Survivin antibody. Survivin immunoprecipitates and inputs (1/10 of IP) were subjected to Western blot analysis with the indicated antibodies. Western blots are representative of three independent experiments.

Given that AURKA had little influence on Survivin mRNA levels, we next explored the underlying mechanism for AURKA-mediated Survivin expression. Depletion of AURKA led to increased Survivin protein degradation following treatment with the translation inhibitor cycloheximide (Figures 4a and b). Indeed, depletion of AURKA reduced Survivin protein half-life, indicating that AURKA stabilized Survivin and regulated Survivin levels via post-translational mechanisms.

As AURKA has been shown to regulate protein ubiquitylation and proteasomal degradation,^10, 26^ we next asked whether AURKA suppressed Survivin polyubiquitylation and proteasomal degradation. We first tested the effects of proteasome inhibitor MG132 in conjunction with AURKA kinase inhibitor or siRNA depletion on Survivin expression. Western blot analysis indicated that AURKA kinase inhibition or siRNA depletion alone reduced Survivin expression, whereas the addition of MG132 could rescue its expression level (Figures 4c and d), suggesting that the Survivin protein downregulation in response to AURKA kinase inhibition or AURKA depletion was at least partially, due to ubiquitin-proteasomal degradation. These findings were corroborated by the *in vivo* Survivin ubiquitylation assay which showed that AURKA knockdown decreased Survivin stability and this was associated with an increase in its polyubiquitylation (Figure 4d).

### AURKA modulates SCF^FBXL7^ to suppress Survivin degradation

**Figure 5:**
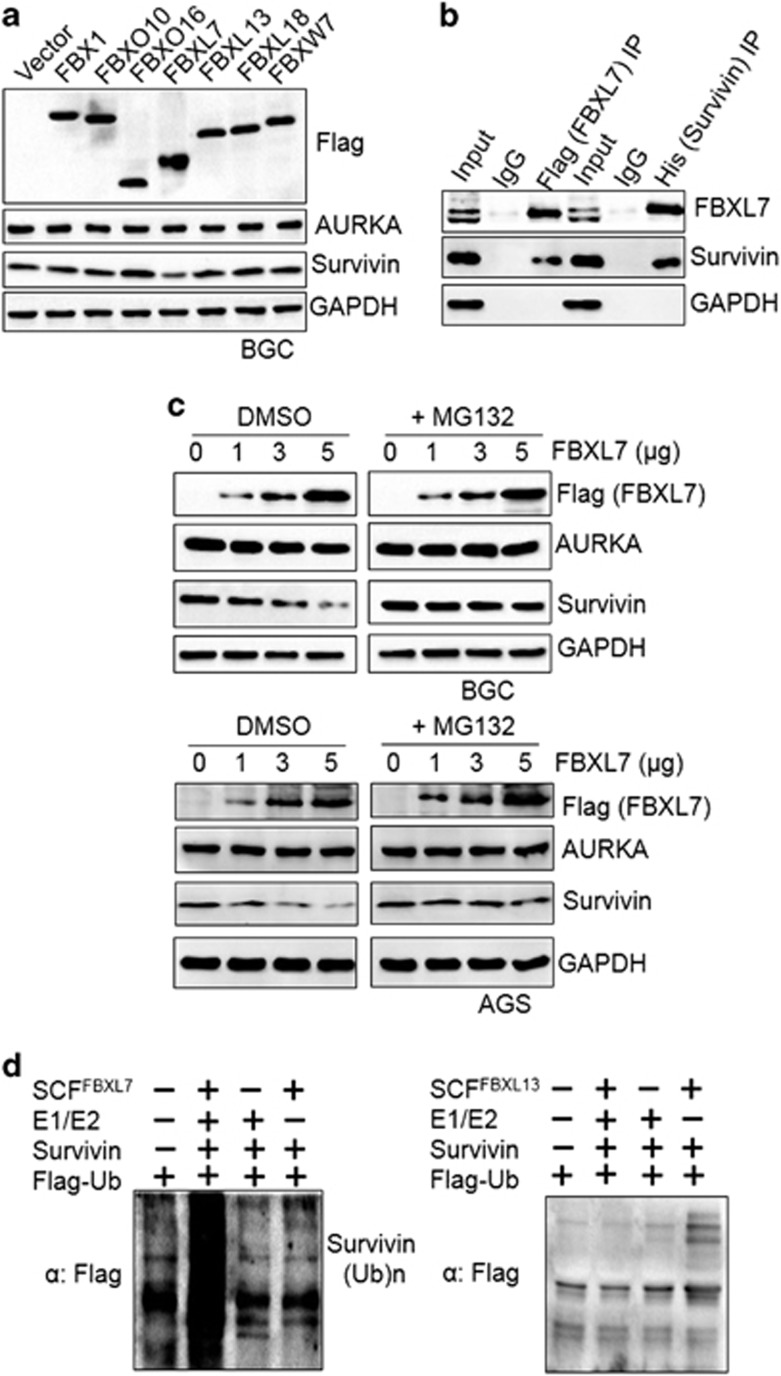
SCF^FBXL7^ targets Survivin for polyubiquitylation and proteasomal degradation. (**a**) Cells were transfected with control plasmid pLVX or Flag-tagged F-box plasmids. After 24 h, cell lysates were prepared and analyzed for Flag, AURKA, Survivin and GAPDH immunoblotting. (**b**) Coimmunoprecipitation (co-IP) assay for determination of FBXL7 and Survivin interaction. HEK293T cells were transfected with Flag-tagged FBXL7 and His-tagged Survivin and cultured for 24 h. Cells were lysed in co-IP buffer followed by immunoprecipitation with rabbit Flag, His and control IgG antibodies. Input (1/25 of IP) and immunoprecipitates were processed for western blotting with the indicated antibodies. (**c**) Cells were transfected with increasing amounts of FBXL7 plasmid. After 24 h, cells were collected and processed for Flag, AURKA, Survivin and GAPDH immunoblotting. (**d**) *In vitro* ubiquitylation of recombinant Survivin with E1, E2, E3-FBXL7 or E3-FBXL13, SKP1, Cul1, RBX1 and ubiquitin-Flag. Upon activation with ATP, samples were incubated at 30 °C for 30 min. The reaction was stopped by addition of SDS–PAGE loading buffer and samples were boiled for 10 min. Polyubiquitylated Survivin was detected by immunoblotting with Flag antibody.

Protein proteasomal degradation precedes orchestrated events involving a series of enzymatic reactions comprising of E1 ubiquitin activating enzyme, E2 ubiquitin conjugating enzymes, and E3 ubiquitin ligases. The E1 and E2 are common enzymes, while E3 ubiquitin ligases are highly substrate specific.^27^ To delineate the E3 ligase responsible for Survivin degradation controlled by AURKA in gastric cancer cells, we performed mass spectrometry analysis of Survivin immunoprecipitates after treatment with MG132 (data not shown) and identified a number of putative Survivin-interacting E3 ubiquitin ligases. To confirm further our mass spectrometry-based data, we selected seven Survivin-interacting proteins with potential E3 ligase activity, and had the genes cloned and transiently expressed in BGC823 cells (Figure 5a). Amongst these E3 ligases, only FBXL7 when overexpressed reduced Survivin expression at the protein level. We then examined Survivin and FBXL7 interaction by transiently co-expressing His-tagged Survivin and Flag-tagged FBXL7 in HEK293T cells followed by coimmunoprecipitation (Figure 5b). We found the Flag-tagged FBXL7 coprecipitated with His-tagged FBXL7 and *vice versa*, but not with the control IgG, suggesting that Survivin interacted with FBXL7 either directly or as part of a larger complex. Next, we transiently transfected AGS and BGC823 cells with increasing amounts of Flag-FBXL7 plasmid (Figure 5c) and found Survivin protein levels decreased in a dose-dependent manner in response to FBXL7 ectopic overexpression. To confirm the specificity of FBXL7 for Survivin, we purified all components of ubiquitylation reaction using the prokaryotic protein expression system and performed *in vitro* ubiquitylation analysis. As indicated in Figure 5d, SCF^FBXL7^ promoted the generation of polyubiquitylated Survivin species, thus confirming that Survivin was a specific substrate of the SCF^FBXL7^ E3 ligase complex.

**Figure 6:**
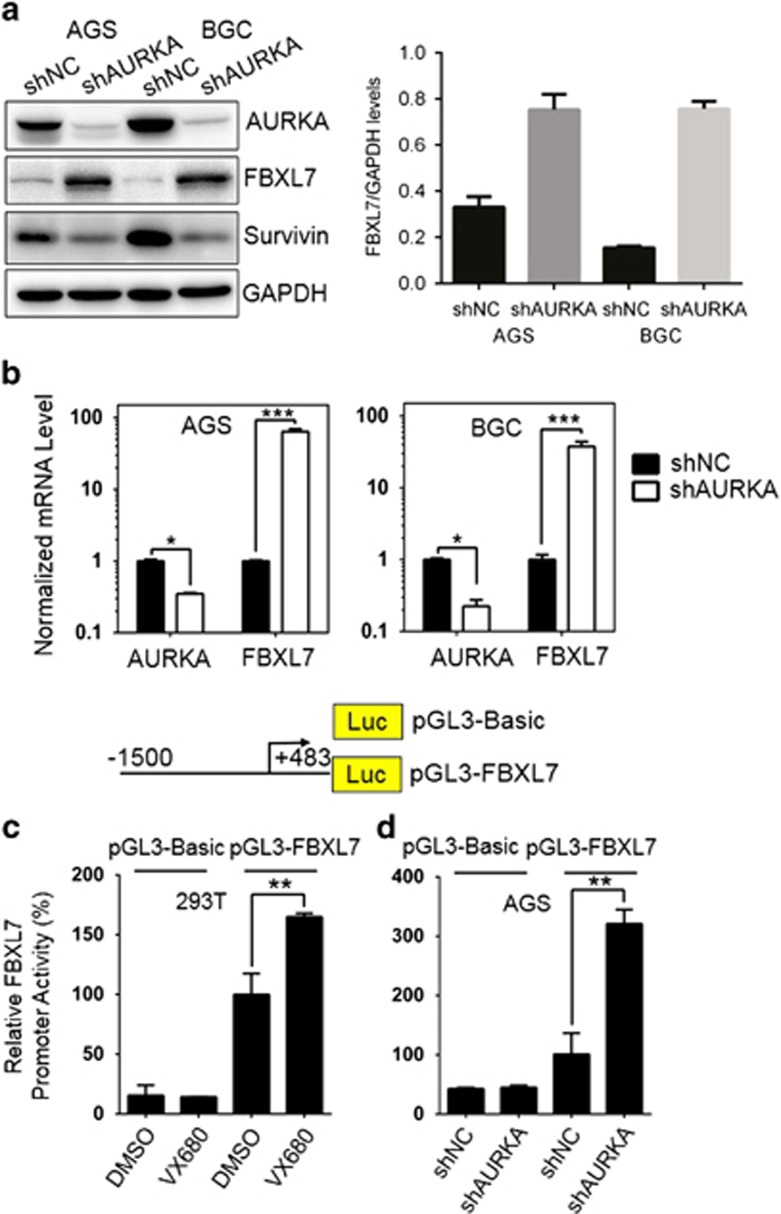
AURKA regulates FBXL7 expression through modulating its promoter activity. (**a**) Western blot and (**b**) quantitative reverse transcription–PCR (qRT–PCR) analysis were performed on AGS and BGC823 cells stably expressing shAURKA or shNC. Cells were cultured in the presence of 1 μg/ml doxycycline. Forty-eight hours after induction, cells were harvested and processed for immunoblotting and qRT–PCR. The experiments were repeated three times independently. Densitometry and qRT–PCR results were normalized against GAPDH protein and mRNA levels, respectively. Data are mean±s.d. of three independent experiment. (**c** and **d**) FBXL7 promoter activity in response to AURKA kinase inhibition and shRNA-mediated AURKA depletion. Promoter activities in response to dimethyl sulfoxide (DMSO) treatment or shNC were set as 100% and other activities are relative to this. Data present means±s.e.m. of three independent experiments and statistical analysis was performed with one-way ANOVA, *P⩽0.05; **P⩽0.01; ***P⩽0.001.

Recent evidence demonstrated that AURKA could regulate c-Myc expression through cooperating with hnRNP K,^8^ we hypothesized that AURKA regulated FBXL7 expression in order to control Survivin steady state. To test this hypothesis, we depleted AURKA in both AGS and BGC823 cells and analyzed FBXL7 expression. As shown in Figure 6a, AURKA depletion significantly increased FBXL7 protein levels. Consistent with the Western blot results, FBXL7 mRNA levels also increased in AURKA knockdown cells (Figure 6b), indicating AURKA regulates FBXL7 expression at the transcriptional level. It is noteworthy that endogenous FBXL7 mRNA and protein levels were low in AURKA wild-type gastric cancer cells; however, both FBXL7 mRNA and protein levels reached detectable levels only after AURKA depletion. To examine AURKA-mediated FBXL7 transcriptional activity, we cloned a 1.5 kb fragment of *FBXL7* upstream of the transcriptional start site into the pGL3 basic vector and tested for its promoter activity. As shown in Figures 6c and d, both total AURKA depletion and kinase activity inhibition significantly increased *FBXL7* promoter activity, suggesting that AURKA suppressed *FBXL7* promoter activity in a kinase-dependent manner.

### FBXL7 suppression by AURKA is dependent on FOXP1 transcriptional activity

**Figure 7:**
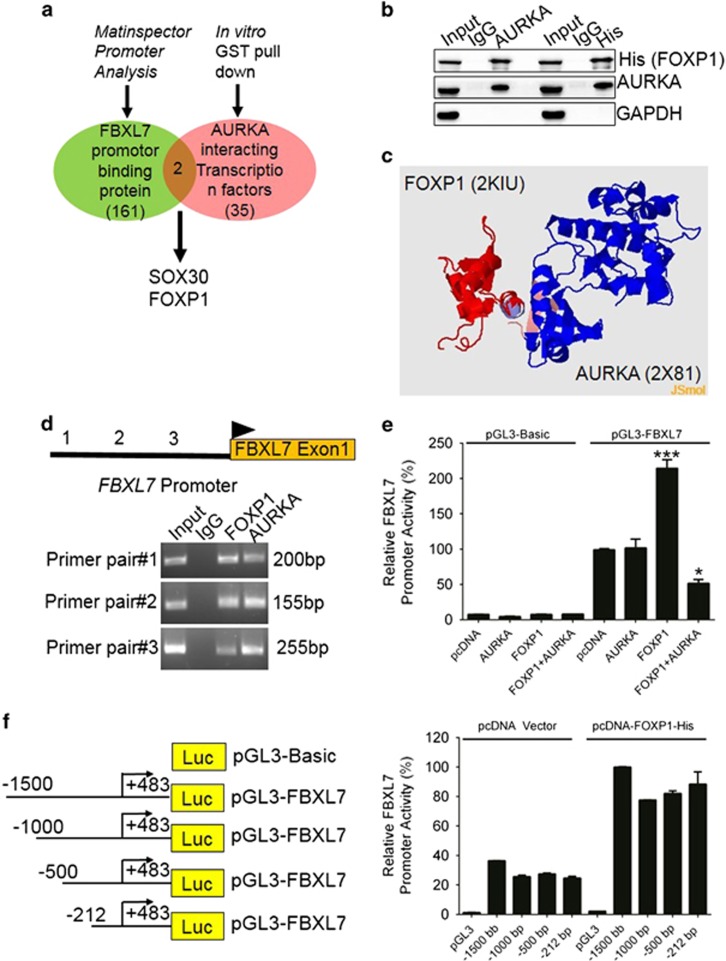
AURKA negatively regulates FOXP1-mediated FBXL7 promoter activity. (**a**) AURKA-interacting transcription factor-like proteins were identified using *in vitro* GST pulldown (red area). FBXL7 promoter-binding proteins were deduced from MatInspector analysis (green area). FOXP1 was present in both data sets and selected for further studies. (**b**) HEK293T cells were transfected with His-tagged FOXP1 and AURKA plasmids and whole-cell lysates co-immunoprecipitated with control IgG, His and AURKA antibodies. Input (1/25 of IP) and immunoprecipitates were processed for immunoblotting with the indicated antibodies. (**c**) *In silico* protein–protein interaction between AURKA and FOXP1. PDB files were obtained from PDB databank (http://www.rcsb.org/pdb) and analyzed for AURKA and FOXP1 interacting residues using PRISM webserver (cosbi.ku.edu.tr/prism/). (**d**) Recruitment of FOXP1 and AURKA to FBXL7 promoter in gastric cancer cells. AURKA and FOXP1 occupancy at the FBXL7 promoter was analyzed by chromatin immunoprecipitation (ChIP) using AURKA, FOXP1 and control IgG antibodies. Input and ChIP DNA was processed for conventional PCR using three different primer pairs, spanning a 2 kb region of FBXL7 promoter. (**e**) Validation of FBXL7 promoter as a direct target of FOXP1. HEK293T cells were transfected with empty pGL3 basic or pGL3-FBXL7 promoter and pcDNA vector, AURKA and FOXP1 as activators. After 24 h, cells were harvested and processed for dual luciferase activity. FBXL7 promoter activity in response to pcDNA vector was set 100% and compared with other samples. Each sample was transfected in triplicates and three repeated experiments were performed in which similar results were obtained. (**f**) The luciferase activities of FBXL7 promoter 5′ deletion constructs in response to pcDNA vector or FOXP1. The −1500 bp FBXL7 promoter activity was set as 100% and compared with −1000, −500 and −212 bp deletion constructs. FOXP1 strongly activated all 5′ deletion constructs affirming that FBXL7 promoter has multiple FOXP1-binding sites. Each sample was transfected in triplicates and three independent experiments were performed in which similar results were obtained. Data present mean±s.e.m. of three independent experiments and statistical analysis was performed with one-way ANOVA, *P⩽0.05; **P⩽0.01; ***P⩽0.001.

To further explore the possible mechanism by which AURKA regulates FBXL7 expression, we examined putative transcription factor binding sites in −1.5 kb to +1 region of the *FBXL7* promoter region using the MatInspector module of the Genomatix database^28^ (Supplementary Table 5). We also performed *in vitro* GST pulldown assay to determine AURKA-interacting transcription factors. For that purpose, we purified GST and GST-AURKA proteins using prokaryotic expression system and incubated them with nuclear and cytoplasmic fractions from AGS cells followed by *in vitro* GST pulldown. The pulldown assay showed that AURKA bound to about 35 proteins with transcription factor activity (Supplementary Table 6). Interestingly, SOX30 and FOXP1 were two factors detected by the pulldown assay and the MatInspector promoter-binding transcription factor analysis (Figure 7a). To confirm the *in vitro* GST-pulldown assay results, we transiently transfected HEK293T cells with His-FOXP1 and AURKA plasmids and performed coimmunoprecipitation studies and found that both AURKA and FOXP1 could pull down each other, but the control antibody did not show any binding (Figure 7b), indicating AURKA and FOXP1 indeed interacted with each other. Furthermore, we confirmed protein–protein interaction between AURKA and FOXP1 using PRISM database,^29, 30^ which predicts the binding site of two proteins using known template interfaces (Figure 7c). PRISM analysis results further revealed that residues 507–519 of FOXP1 located in forkhead domain might be involved in interacting with residues 127–209 of AURKA (data not shown). Moreover, mass spectrometry analysis of *in vitro* AURKA phosphorylated FOXP1 (data not shown) identified S83, S104 and S440 FOXP1 (Supplementary Figure 3d) as putative AURKA phosphorylated sites on FOXP1. Intriguingly, one of them (S440) was located within the transactivation domain of FOXP1.^31^ It was therefore conceivable that AURKA might target FOXP1 for phosphorylation and transcriptional repression.

We then investigated the *in vivo* recruitment of FOXP1 to the *FBXL7* promoter by chromatin immunoprecipitation (ChIP). As shown in Figure 7d, both FOXP1 and AURKA antibodies could effectively immunoprecipitate the *FBXL7* promoter DNA, indicating both FOXP1 and AURKA, are recruited to the *FBXL7* promoter directly or as part of a protein complex. Next, we examined whether FOXP1 binding to *FBXL7* promoter enhanced *FBXL7* gene expression. To this end, we performed luciferase reporter assay in HEK293T cells using full-length wild-type *FBXL7* promoter (Figure 7e). The reporter assay showed that AURKA expression did not affect *FBXL7* promoter activity, while FOXP1 increased considerably the *FBXL7* promoter activity. Notably, the expression of AURKA also significantly repressed the induction of FBXL7 promoter activity by FOXP1, suggesting AURKA modulated FOXP1 activity to restrict FBXL7 expression, whereas in the absence of AURKA, FOXP1 served as an activator of *FBXL7* expression. To explore further the ability of FOXP1 to *trans*activate the *FBXL7* promoter, we generated *FBXL7* promoter 5′-truncation constructs to test their ability to be transactivated by FOXP1 (Figure 7f). The promoter/reporter assays showed that FOXP1 induced the promoter activity of all 5′ deletion constructs, confirming that FOXP1 could induce FBXL7 expression through its promoter. Moreover, the results also strengthened the MatInspector database findings that FOXP1 bound to multiple sites on the *FBXL7* promoter. In addition, we also determined whether these transcriptional effects were mediated at the translational level. We transfected AGS cells with the empty pcDNA vector, AURKA, FOXP1, or FOXP1 and AURKA together, and investigated the FBXL7 protein levels by immunoblotting. As shown in Supplementary Figure 3a, ectopic expression of AURKA significantly downregulated FBXL7 protein levels, while FOXP1 alone upregulated FBXL7 expression. However, a combination of AURKA and FOXP1 again suppressed FBXL7 expression, indicating that AURKA negatively regulated FBXL7 expression through modulating FOXP1. Together, these results suggest that AURKA targets FOXP1 to negatively regulate the expression of FBXL7, which can in turn negatively regulate Survivin protein levels in gastric cancer cells.

## Discussion

Mitotic kinases, including AURKA, are key signaling components of genotoxic response pathways.^32^ Previous studies have documented aberrant expression of AURKA in various carcinomas and hematological malignancies.^7, 33^ In addition, our earlier data has also suggested that AURKA was highly expressed in epirubicin resistance breast tumor initiating cells and contributed to the maintenance of stemness and drug resistance in breast cancer.^34^ Given the essential roles of AURKA overexpression in cancer progression, targeting AURKA offers an attractive approach for cancer therapy. In the present study, we found that AURKA could override DNA-damaging agent-induced cell death in gastric cancer cell lines, resulting in the development of drug resistance which was at least partially mediated through Survivin stabilization.

Gastrointestinal adenocarcinoma responds poorly to conventional chemotherapy and has an unfavorable outcome if diagnosed in an advanced stage.^35^ Therefore, it is vital to identify early prognostic biomarkers and effective therapeutic targets for gastric adenocarcinoma management. A number of epidemiological studies have assessed AURKA expression in gastric cancer.^9, 36, 37, 38^ For example, in an analysis of 88 human primary gastric tumor specimens, Kamada *et al.* reported positive staining for AURKA in 41% of samples.^9^ Similarly, in an analysis of 130 gastric cancer subjects, >50% AURKA positivity was reported in upper gastrointestinal adenocarcinoma.^36^ In the present study, we analyzed a large cohort of 240 gastric cancer patient specimens and found that AURKA was highly overexpressed in gastric cancer tissues as assessed by immunohistochemistry and western blotting (Figure 1a). Moreover, immunohistochemical analysis showed that AURKA overexpression was correlated with tumor stage, lymph node metastasis and distant metastasis, but not with gender, age, tumor size, tumor site and tumor grading, suggestive of a role of AURKA in gastric cancer progression. In agreement, high AURKA expression was inversely associated with overall survival of gastric cancer patients (Figure 1b) and had been shown to be an independent prognostic factor for gastric cancer (Figure 3c). This was consistent with our previous study in laryngeal squamous cell carcinoma patients that elevated AURKA expression predicted poor overall survival.^39^ Resistance to DNA-damaging agent-induced apoptosis is a major mechanism of poor chemotherapeutic response. Our study indicated that the limited chemotherapy efficacy might be due to high expression levels of AURKA in gastric cancer. Consistent with this notion, overexpression of AURKA in gastric cells could overcome DNA damage-induced apoptotic cell death (Figure 1d), which again was in agreement with another previous study.^32^ Recent reports showed that AURKA overexpression was essential for the tumorigenic capacity and drug resistance of breast tumor initiating cells^34^ as well as chemoresistance in lung cancer cells,^40^ supporting our study that deregulated overexpression of AURKA in gastric cancer led to clinical chemoresistance.

As a member of IAPs, Survivin can confer drug resistance and is correlated with drug refractory tumors.^18, 19, 41, 42^ Survivin expression is significantly upregulated in gastric cancers compared with the tissues of normal mucosa, atrophic gastritis and intestinal metaplasia, and is negatively associated with OS of patients who received CDDP-based chemotherapy.^43^ Mechanistically, Survivin is upregulated by upstream factors, such as p34/cdc2-cyclin B1 and Plk1.^44, 45^ In the present work, we demonstrated that inhibition of AURKA kinase activity by VX-680 or depletion of total AURKA, suppressed Survivin expression, whereas overexpression of AURKA upregulated Survivin in gastric cancer cells (Figures 2a and b). These findings are further corroborated by the finding that AURKA and Survivin co-expressed in gastric cancer patients' specimens (Figure 2d). In addition, we also found that AURKA regulated Survivin expression through suppression of its ubiquitylation and proteasomal degradation (Figure 4). In previous reports, AURKA has been shown to stabilize LIMPK2 in breast cancer^46^ and N-myc in neuroblastoma^26^ by inhibiting their ubiquitylation and degradation.

In dissecting molecular mechanism that leads to Survivin upregulation in response to AURKA overexpression, we found that Survivin upregulation by AURKA was not regulated at the transcriptional level (Supplementary Figure 2). Previous studies have shown that p53 represses Survivin transcription through promoter hypermethylation.^47^ In our studies, VX-680 significantly suppressed Survivin protein levels in both the wild-type and p53 knockout MEFs, indicating the regulation of Survivin expression by AURKA was unlikely to be dependent on p53.

**Figure 8:**
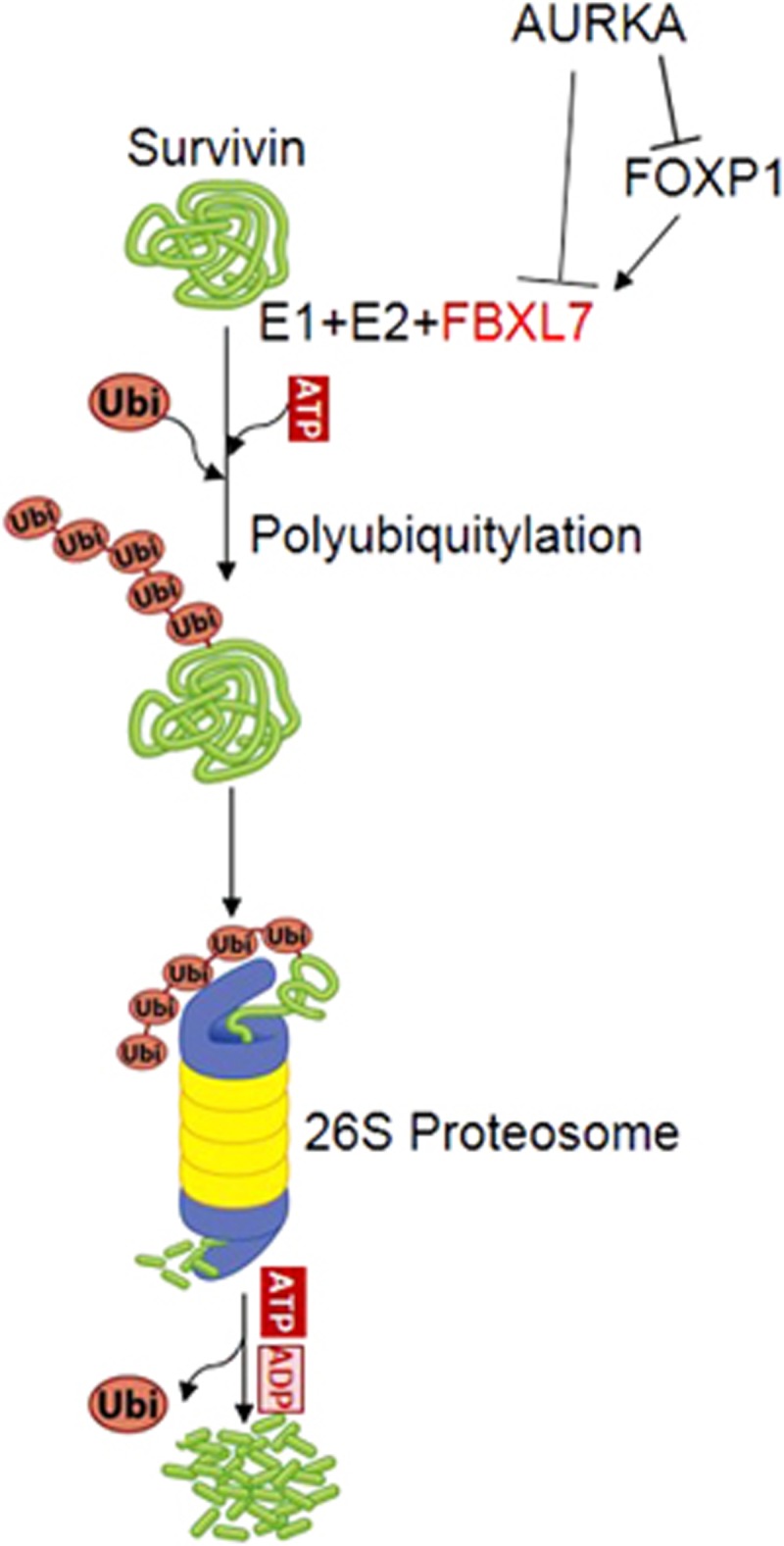
Schematic model of a proposed mechanism showing how AURKA suppresses Survivin protein degradation. Survivin undergoes proteasomal degradation mediated by SCF^FBXL7^ E3 ligase. However, AURKA downregulates FBXL7 expression through modulation of FOXP1. Consequently, Survivin is stabilized and have an important role in gastric cancer cell survival and drug resistance.

Survivin stabilization and upregulation by post-translational modifications have been described previously.^48, 49^ In a mass spectrometry analysis of Survivin co-immunoprecipitates, we found FBXL7 E3 ubiquitin ligase acted as a Survivin-interacting protein. FBXL7 belongs to the leucine-rich repeats containing F-box family of proteins and is a part of SCFs (SKP1-Cul1-F-box) E3 ligase complex. FBXL7 has been shown to induce the ubiquitylation of AURKA during mitosis^50^ and Survivin in a cell cycle-independent manner.^48^ FBXL7 is a potential tumor suppressor gene as a previous study found an association between SNPs in *FBXL7* and an increased breast cancer risk.^51^ We showed that in unsynchronized cells, FBXL7 promoted Survivin degradation by proteasomal pathway in our experimental setting, which was in agreement with a previous report,^48^ while AURKA expression remained unaffected in response to FBXL7 overexpression. Indeed, we found that both the FBXL7 mRNA and protein levels increased in response to AURKA depletion in both AGS and BGC823 cells. Basal levels of FBXL7 mRNA and protein were low in gastric cancer cells and only became detectable after AURKA depletion. This led us to speculate that FBXL7 was a potential tumor suppressor gene and its expression was repressed by AURKA in gastric cancer. We also found the FOXP1 transcription factor to be an AURKA-interacting partner in the regulation of FBXL7 transcription. This finding was in agreement with a previous ChIP-sequencing analysis that FOXP1 bound to the *FBXL7* promoter *in vivo*.^52^ In concordance, we found that FOXP1 could activate *FBXL7* promoter activity, while AURKA repressed the FOXP1-mediated induction in *FBXL7* promoter activity. Our *In silico* analysis showed that FOXP1 was a potential AURKA substrate that led us to speculate that AURKA could post-translationally modify FOXP1 to alter its transcriptional activity. Since AURKA depletion did not affect FOXP1 cytoplasmic/nuclear localization and shuttling, we concluded that AURKA modulated FOXP1 without affecting its (Supplementary Figure 3b and 3c) translocation. Collectively, our data suggested that AURKA negatively regulated FBXL7 expression through modulating the activity of the FOXP1 transcription factor and thereby, restricting Survivin degradation by the FBXL7-ubiquitinylation complex (Figure 8).

Notably, inhibition of AURKA using the inhibitor VX-680 limited cell proliferation and that VX-680 synergized with the DNA-damaging agent doxorubicin in suppressing cell proliferation in BGC823 cells (Figure 3a). These results are concordant with previous findings that AURKA suppression increased chemosensitivity to docetaxel in both *in vitro* and *in vivo* models of esophageal squamous cell carcinoma;^53^ however, the mechanism underlying the AURKA-mediated chemotherapeutic resistance remains enigmatic. Previous studies have shown that Survivin is upregulated because of its cytoprotective function in response to several anti-cancer agents, including doxorubicin, suggesting that tumor cells enhance Survivin expression to counteract the apoptotic signals induced by chemotherapeutic agents.^42, 54^ In our work, we found that doxorubicin alone induced Survivin expression, while the combination of VX-680 and doxorubicin induced apoptotic cell death synergistically in the human gastric BGC823 cells, accompanied by a decrease in AURKA activation and Survivin expression (Figure 3b). Collectively, our data suggest that the synergistic cytotoxic effect of AURKA inhibition and doxorubicin is possibly attributable to a convergence of signals that ultimately lead to the downregulation of the expression of the anti-apoptotic protein Survivin. This data proposes the inclusion of AURKA inhibitors as therapeutic agents for gastric cancer management. In conclusion, our data suggest that AURKA limits Survivin ubiquitylation and degradation in gastric cancer and provide a novel therapeutic target to promote the efficacy of DNA-damaging agent in gastric cancer.

## Materials and Methods

### Cell culture, plasmids and transfections

Gastric cell lines AGS and BGC823 were cultured in RPMI 1640 (Gibco, Carlsbad, CA, USA) HEK293T and mouse embryonic fibroblast (MEF) were cultured in Dulbecco's modified eagle medium (DMEM) (Gibco). Cell culture media were supplemented with 10% heat-inactivated fetal bovine serum (Gibco), 100 U/ml ampicillin and 100 μg/ml streptomycin (Gibco). Treatments with doxorubicin (Sigma-Aldrich, St Louis, MO, USA), doxycycline (Clonetech, Shiga, Japan), VX-680 (Selleck Chemicals, Kava Technology), cycloheximide (Amresco, Solon, OH, USA) and MG132 (Sigma-Aldrich) were carried out as indicated. Cells were transfected using lipofectamine 2000 transfection reagent (Invitrogen, Carlsbad, CA, USA). For details, see Supplementary Materials and Methods.

### Patients and clinical tissue specimens

See Supplementary Materials and Methods

### Immunoblotting and immunoprecipitation

Cells, gastric cancer tissues and paired normal adjacent tissues were lysed on ice with RIPA buffer. For further details, see Supplementary Materials and Methods. Detailed information about the antibodies used in this study is listed in Supplementary Table 4.

### Immunofluorescence staining

Immunofluorescence staining was performed as described.^55^ For details, see Supplementary Materials and Methods.

### Immunohistochemical staining

Both staining intensity and extent were included to evaluate AURKA or Survivin expression. Evaluation was done by at least two independent pathologists. Moderate or strong cytoplasm staining was considered as positive reaction. In analysis, specimen was determined as high staining when >30% cells showed visible brown granules.

### Cell survival (MTT) assay

Cells were seeded into 96-well flat bottom plates and exposed to increasing doses of VX-680 (Kava Technology, San Diego, CA, USA), doxorubicin (Sigma-Aldrich) separately or combination. Standard 3-(4,5-Dimethylthiazol-2-yl)-2, 5-diphenyltetrazolium bromide (MTT) assay was performed.

### TUNEL assay

Cells were seeded in a 6-well plate, collected and fixed with 4% paraformaldehyde (Sigma-Aldrich) for 30 min. Cells were then labeled by TdT-mediated dUTP Nick-End Labeling Kit (BD Biosciences, San Jose, CA, USA) and analyzed on a Beckon Dickinson FACScan.

### Evaluation of drug interactions

The interaction between VX-680 and doxorubicin was analyzed to determine whether the combination was additive or synergistic. This program is based on the Jin's method, which is performed based on the following equation: *q*=D1+2/(D1+D2−D1 × D2), where D1+2 indicates the effect when cells were used in combination with drug 1 and 2, and D1 or D2 indicates the effect when used alone.

### GST pulldown for identifying interacting transcription factors with AURKA

To identify transcription factors that interact with AURKA, 500 μg AGS cells nuclear extract, prepared as described previously.^8^ In-gel digestion and recovery of peptides were performed as described earlier.^56^ For details, see Supplementary Materials and Methods.

### *In vitro* ubiquitylation assay

The *in vitro* ubiquitylation assay was performed in a volume of 50 μl containing 50 mm Tris pH 7.6, 0.5 m DTT, 5 mm MgCl_2_, 2 mm ATP, 50 nm E11, 0.5 μm UbcH5B, 0.5 μm UbcH5C, 0.5 μm UbcH7, 2 μm ubiquitin-Flag, 20 nm Rbx1, 20 nm Cul1, 20 nm Skp1, 20 nm FBXL7, 200 nm Survivin at room temperature for 1 h. Reaction was stopped by addition of SDS–PAGE loading buffer and Survivin ubiquitylation visualized by immunoblotting with Flag antibody.

### ChIP assay

ChIP assay was performed as previously described.^57^ For details, see Supplementary Materials and Methods.

### RNA extraction and RT–qPCR assays

Total RNA was prepared using TRIzol (Invitrogen), according to manufacturer's instructions. For details, see Supplementary Materials and Methods. Primers were purchased from Invitrogen and primer sequences are listed in Supplementary Table 4.

### *In silico* protein–protein interaction

Tertiary-structure-based *in silico* protein–protein interaction between AURKA and FOXP1 was determined by PRISM method which is based on template matching with known protein structures.^29, 30^ We obtained PDB files; 2 × 81 (AURKA) and 2KIU (FOXP1) as a target set of proteins and analyzed their predicted protein–protein interaction as described elsewhere.^58^

### Promoter assay

Promoter dual luciferase assays (Promega, Madison, WI, USA) were performed as per manufacturer's instructions. For details, see Supplementary Materials and Methods.

### Statistical analysis

Statistical analysis was performed using SPSS v. 16.0 (SPSS, Chicago, IL, USA). All *P*-values quoted were two-sided. *P*<0.05 was considered statistically significant.
