# Supplementary material for: Aurora kinase A regulates Survivin stability through targeting FBXL7 in gastric cancer drug resistance and prognosis
Source: Oncogenesis. 2017 Feb 20;6(2):e298–. doi: 10.1038/oncsis.2016.80 (PMC5337621; doi:10.1038/oncsis.2016.80)
Supplement: Supplementary Figure Legends [file oncsis201680x1.doc]

**Supplementary Figure S1** Doxorubicin induces DNA damage in gastric cancer cells. BGC823 cells were incubated with 0.5 µg/ml doxorubicin for 24 hours and subjected to immunofluorescence staining with phospho-Histone H2A.X (S139) antibody and immunoblotting with the indicated antibodies. Representative result is shown.

**Supplementary Figure S2** AURKA does not regulate survivin transcription in BGC823 cells. (**a**) qRT-PCR analysis of AGS and BGC823 cells stably expressing shAURKA or shNC as in Figure 2a. The experiments were repeated three times independently and results normalized to GAPDH mRNA levels. Data shown are means ± s.e.m. of three independent experiment. (**b**) Survivin promoter activity in response to shRNA-mediated AURKA depletion. Promoter activities in response to shNC were set as 100% and other activities shown were relative to this and data was analyzed using one-way ANOVA. Significance: **P≤0.05; **P≤0.01; ***P≤0.001*. (**c**) and (**d**) Western Blot analysis of p53 wild-type and knockout mouse embryonic fibroblasts. Cells were treated with DMSO or AURKA small molecule inhibitor VX-680 for 24 hours followed by processing cell lysates for immunoblotting with the indicated antibodies. Densitometry results were normalized against GAPDH protein levels. Data are mean ± s.e.m. of three independent experiment.

**Supplementary Figure 3** AURKA deregulates FOXP1 activity by phosphorylation but it does not influence FOXP1 cytoplasmic/nuclear shuttling. (**a**) AGS cells were transfected with expression vector pcDNA, AURKA, FOXP1, or FOXP1 and AURKA together. Twenty-four hours after transfection, cell lysates were prepared and processed for immunoblotting with the indicated antibodies. (**b**) AGS cells, as in Figure 2a, were cultured on glass coverslips and stained with rabbit anti-FOXP1 (1:100 dilution) and mouse anti-AURKA (1:100 dilution) as primary antibodies and visualized with Alexa-Fluor anti-rabbit and FITC anti-mouse secondary antibodies. DAPI (1:10000) was used to stain nuclei and cells were visualized in a fluorescence microscope. (**c**) AGS cells as grown in Figure 2a, were fractionated into cytoplasmic and nuclear fractions and processed for immunoblotting with the indicated antibodies. (**d**) Schematic diagramme of FOXP1 structure with Forkhead, Zing finger and coiled coil domains. AURKA phosphorylation sites on FOXP1 determined by mass spectrometry, are indicated as circles.
