# Supplementary figures and images for: Aurora kinase A regulates Survivin stability through targeting FBXL7 in gastric cancer drug resistance and prognosis
Source: Oncogenesis. 2017 Feb 20;6(2):e298–. doi: 10.1038/oncsis.2016.80 (PMC5337621; doi:10.1038/oncsis.2016.80)

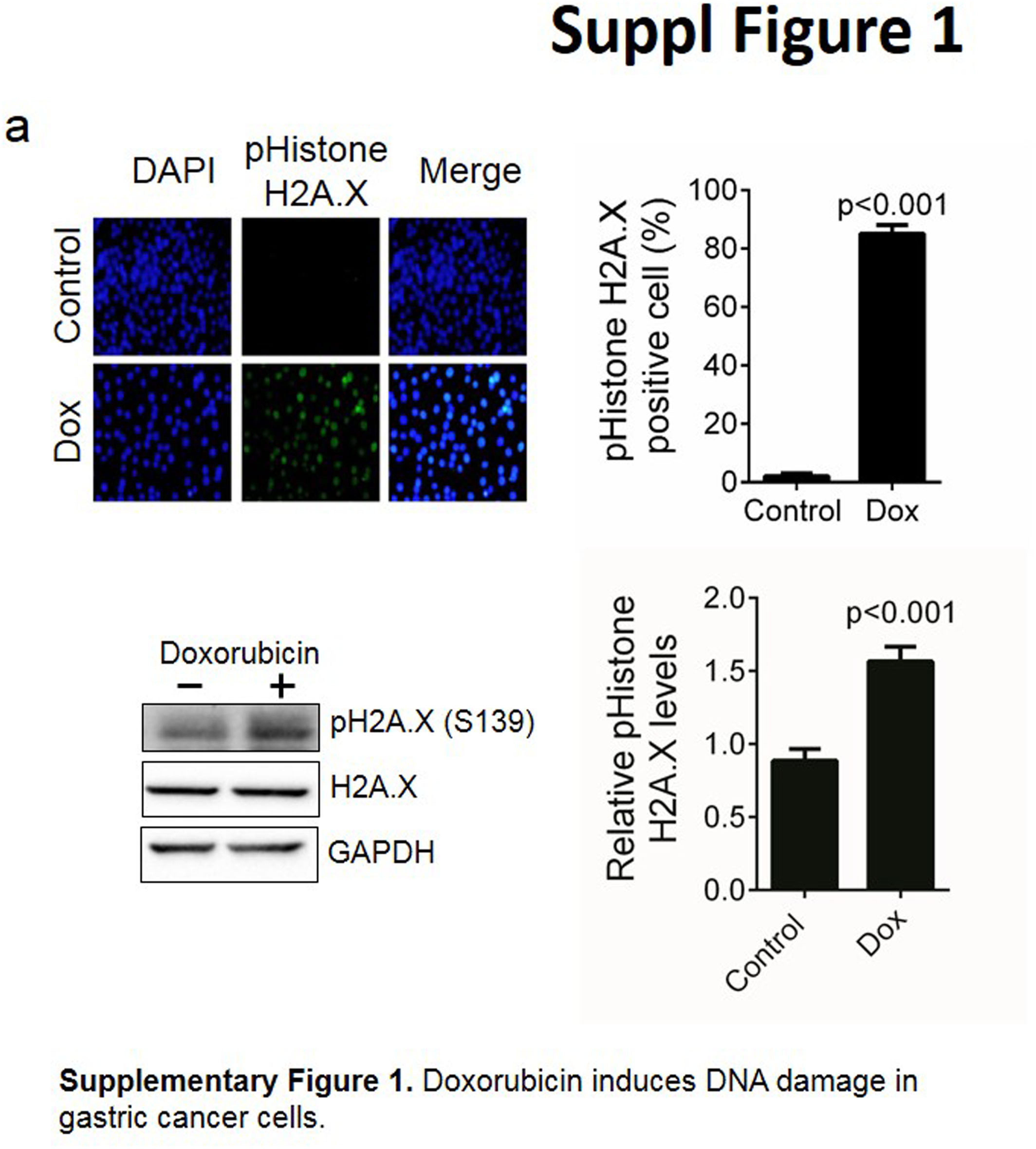

Supplement: Supplementary Figure S1 [file oncsis201680x2.tif]

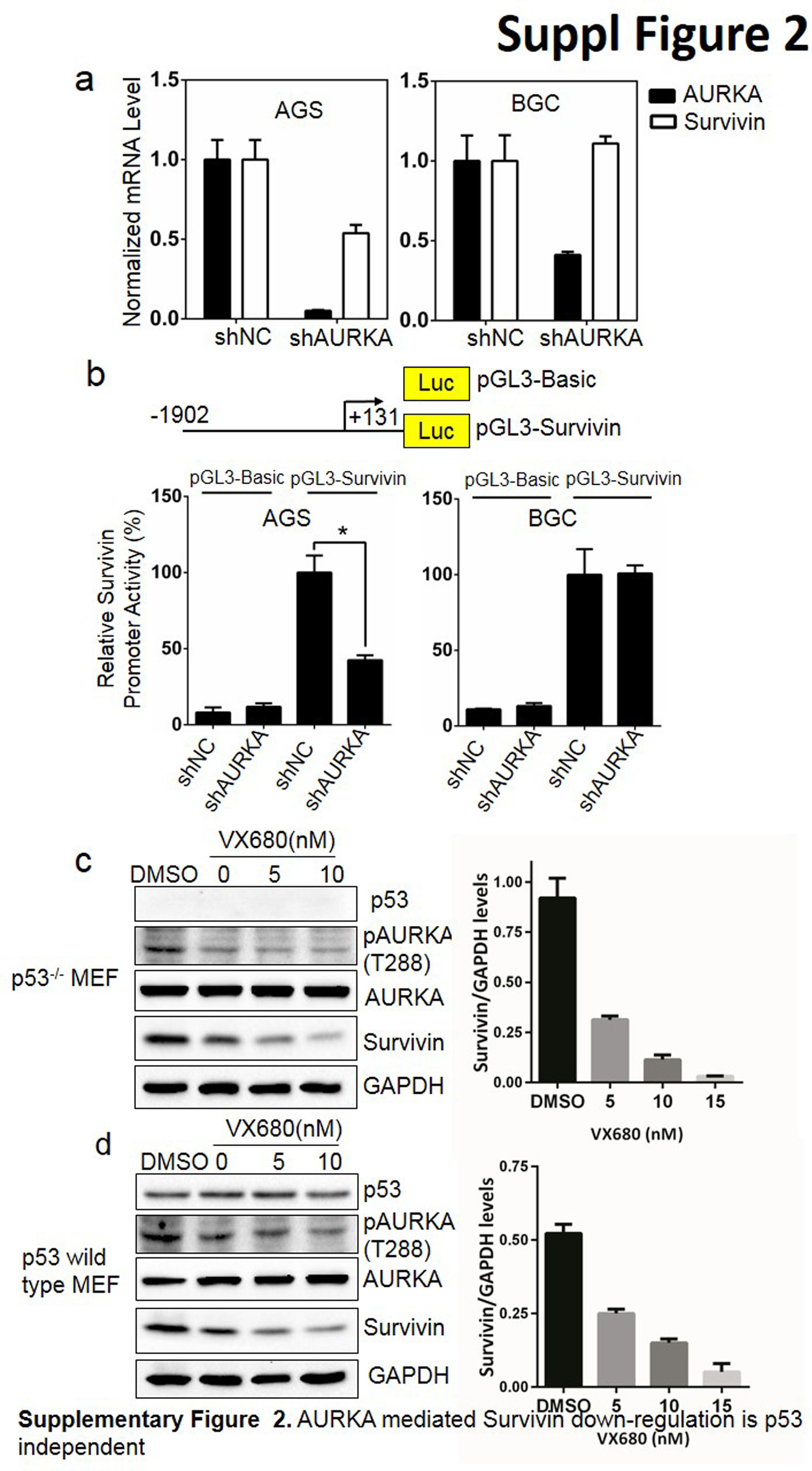

Supplement: Supplementary Figure S2 [file oncsis201680x3.tif]

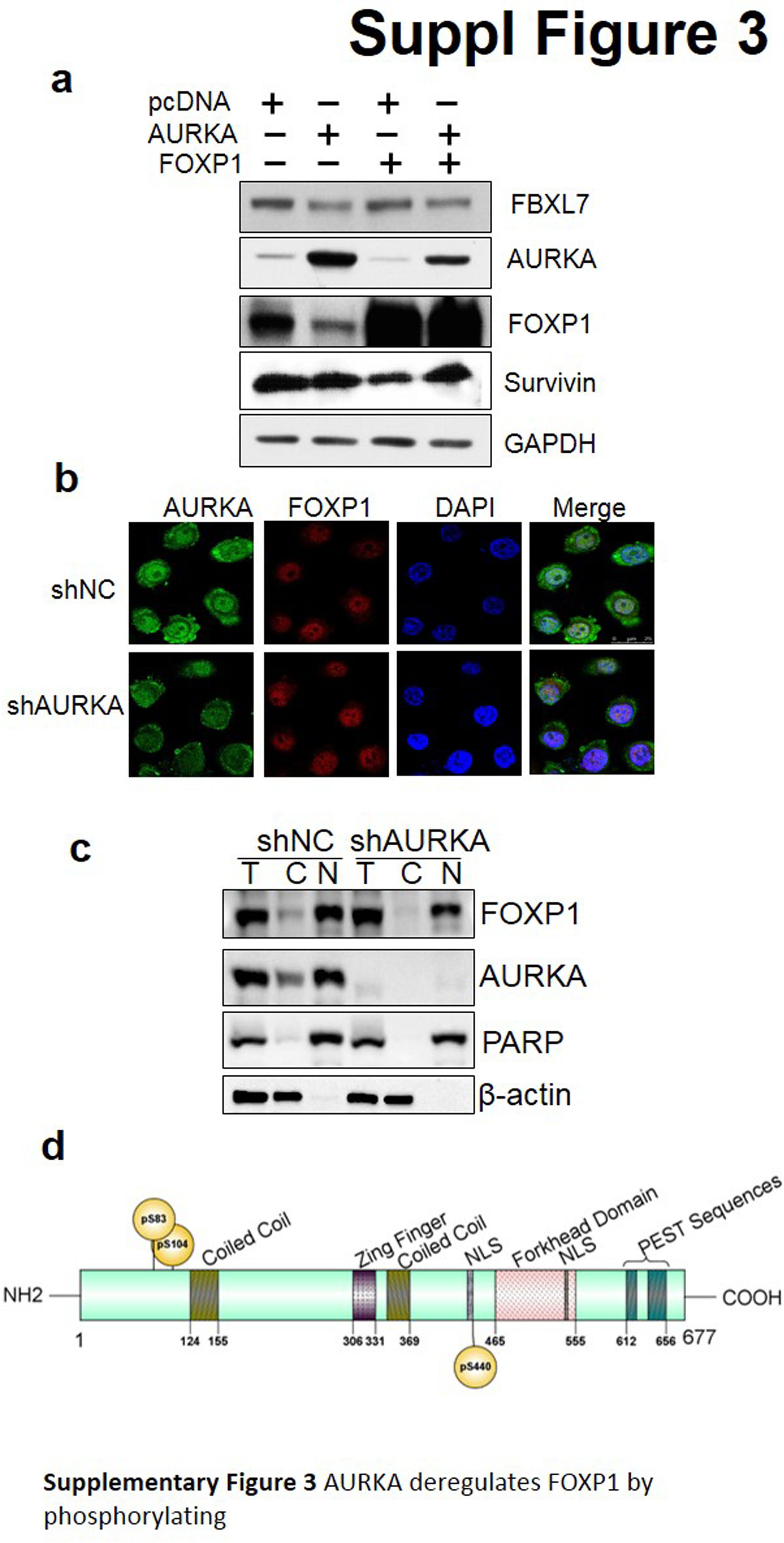

Supplement: Supplementary Figure S3 [file oncsis201680x4.tif]
