# Supplementary material for: Aurora kinase A regulates Survivin stability through targeting FBXL7 in gastric cancer drug resistance and prognosis
Source: Oncogenesis. 2017 Feb 20;6(2):e298–. doi: 10.1038/oncsis.2016.80 (PMC5337621; doi:10.1038/oncsis.2016.80)
Supplement: Supplementary Table 1 [file oncsis201680x5.pdf]

Supplementary Table 1

Clinicopathologic Correlation of AURKA Expression in Gastric Cancer

**Factors**	**All patients**	**No. of patients (%)**		***P***
	***N*=240(%)**	**AURKA low**	**AURKA high**	
Age (y)				0.308
＜60	129 (53.75)	33 (48.5)	96 (55.8)	
≥60	111 (46.25)	35 (51.5)	76 (44.2)	
Sex				0.302
Male	167 (69.6)	44 (64.7)	123 (71.5)	
Female	73 (30.4)	24 (35.3)	49 (28.5)	
Site				0.556
Upper	95 (39.6)	30 (44.1)	65 (37.8)	
Middle	34 (14.2)	8 (11.8)	26 (15.1)	
Lower	108 (45.0)	30 (44.1)	78 (45.3)	
Diffuse	3 (1.2)	0 (0)	3 (1.8)	
Tumor size				0.246
≤4cm	75 (31.3)	25 (36.8)	50 (29.1)	
＞4cm	165 (68.7)	43 (63.2)	122 (70.9)	
Grading				0.954
Well differentiated	2 (0.8)	1(1.5)	1 (0.6)	
Moderated differentiated	54 (22.5)	16 (23.5)	38(22.1)	
Poor differentiated	144 (60.0)	39 (57.4)	105 (61.0)	
Undifferentiated	7 (2.9)	2 (2.9)	5 (2.9)	
Unknow	33 (13.8)	10 (14.7)	23 (13.4)	
Pathologic T classification				0.006
T1	16 (6.7)	5 (7.4)	11 (6.4)	
T2	45 (18.7)	19 (27.9)	26 (15.1)	
T3	135 (56.3)	40 (58.8)	95 (55.2)	
T4	44 (18.3)	4 (5.9)	40 (23.3)	

Pathologic N status				0.007
N negative	69 (28.7)	28 (41.2)	41 (23.8)	
N positive	171 (71.3)	40 (58.8)	131 (76.2)	
Metastasis				<0.0001
No	209 (87.1)	68 (100.0)	141 (82.0)	
Yes	31 (15.2)	0 (0.0)	31 (18.0)	
Pathologic stage (pTNM)				0.002
Ⅰ	29 (12.1)	13 (19.1)	16 (9.3)	
Ⅱ	59 (24.6)	21 (30.9)	38 (22.1)	
Ⅲ	105 (43.7)	30 (44.1)	75 (43.6)	
Ⅳ	47 (19.6)	4 (5.9)	43 (25.0)	
