# Supplementary material for: Aurora kinase A regulates Survivin stability through targeting FBXL7 in gastric cancer drug resistance and prognosis
Source: Oncogenesis. 2017 Feb 20;6(2):e298–. doi: 10.1038/oncsis.2016.80 (PMC5337621; doi:10.1038/oncsis.2016.80)
Supplement: Supplementary Table 2 [file oncsis201680x6.pdf]

Supplementary Table 2

Univariate Analysis of Disease-free and Overall Survival in Gastric Cancer

Characteristic	No.	HR	Disease-free suivival 95%CI	*P*	HR	Overall survival 95%CI	*P*
Age（y）				0.355			0.404
＜60	129	1.00			1.00		
⩾60	111	0.866	0.637-1.176		0.875	0.640-1.197	
Sex				0.567			0.569
Male	167	1.00			1.00		
Female	73	1.101	0.792-1.531		1.103	0. 787-1.544	
Tumor size				<0.001			<0.001
⩽4cm	75	1.00			1.00		
＞4cm	165	2.200	1.518-3.187		2.099	1.440-3.058	
Site				0.021			0.029
Upper	95	1.00			1.00		
Middle	34	0.896	0.560-1.434	0.646	0.971	0.604-1.561	0.904
Lower	108	0.795	0.571-1.108	0.176	0.837	0.596-1.176	0.306
Diffuse	3	4.738	1.462-15.361	0.010	4.899	1.509-15.905	0.008
Grading				0.967			0.941
Well	2	1.00			1.00		
Moderated	54	2951.534	0.000-1E+034	0.825	0.000	0.000-2E+138	0.948
Poor	144	3140.152	0.000-2E+034	0.823	0.951	0.555-1.629	0.855
Undifferentiated	7	2243.345	0.000-1E+034	0.831	1.084	0.677-1.737	0.737
Unknow	33	3117.623	0.000-2E+034	0.824	0.794	0.273-2.315	0.673
Pathologic T classification				<0.0001			<0.0001
T1	16	1.00			1.00		
T2	45	4.864	1.140-20.749	0.033	4.558	1.065-19.503	0.041
T3	135	9.943	2.450-40.353	0.001	9.049	2.228-36.744	0.002

T4	44	27.417	6.595-113.979	<0.0001	24.134	5.811-100.242	<0.0001
Pathologic N status				<0.0001			<0.0001
Negative	69	1.00			1.00		
Positive	171	3.245	2.143-4.914		2.969	1.959-4.502	
Metastasis				<0.0001			<0.0001
No	209	1.00			1.00		
Yes	31	3.865	2.571-5.812		3.902	2.593-5.873	
pTNM stage				<0.0001			<0.0001
Ⅰ	29	1.00			1.00		
Ⅱ	59	2.652	1.162-6.056	0.021	2.624	1.150-5.992	0.022
Ⅲ	105	5.487	2.532-11.888	<0.0001	4.854	2.236-10.536	<0.0001
Ⅳ	47	13.865	6.183-31.093	<.0001	12.196	5.449-27.300	<0.0001
AURKA				0.001			<0.001
Negative	68	1.00			1.00		
Positive	172	1.907	1.317-2.760		1.996	1.360-2.931	
