# Supplementary material for: Aurora kinase A regulates Survivin stability through targeting FBXL7 in gastric cancer drug resistance and prognosis
Source: Oncogenesis. 2017 Feb 20;6(2):e298–. doi: 10.1038/oncsis.2016.80 (PMC5337621; doi:10.1038/oncsis.2016.80)
Supplement: Supplementary Table 3 [file oncsis201680x7.pdf]

Supplementary Table 3
Correlation Between AURKA and Survivin Expression

		AURKA	Survivin
AURKA	Pearson Correlation	1	0.402(**)
	Sig. (2-tailed)		0.001
	N	62	62
Survivin	Pearson Correlation	0.402(**)	1
	Sig. (2-tailed)	0.001	
	N	62	62

** Correlation is significant at the 0.01 level (2-tailed).
