# Supplementary material for: Aurora kinase A regulates Survivin stability through targeting FBXL7 in gastric cancer drug resistance and prognosis
Source: Oncogenesis. 2017 Feb 20;6(2):e298–. doi: 10.1038/oncsis.2016.80 (PMC5337621; doi:10.1038/oncsis.2016.80)
Supplement: Supplementary Table 4 [file oncsis201680x8.pdf]

Supplementary Table 4. List of antibodies used in this study.

S#	Protein	Size (kDa)	Vendor	Dilution
1	Aurora A	48	Upstate Cell Signaling Solutions	1:6000
2	Aurora B	40	Cell Signaling Technology	1:1000
3	Beta Actin	45	Proteintech	1:10000
4	Alpha tubulin	52	Proteintech	1:5000
5	GAPDH	37	Kangchen China	1:10000
6	Survivin	16	Proteintech	1:1000
7	Phospho-Aurora A (Thr288)	48	Cell Signaling Technology	1:500
8	Ubiquitin C		Proteintech	1:1000
9	FOXP1	75	Proteintech	1:2000
10	FBXL7	55	GeneTex	1:500
11	p53	53	Proteintech	1:1000
12	PARP	116, 89	Cell Signaling Technology	1:5000
13	Anti-phospho Serine/threonine		ECM Biosciences	1:1000
14	Anti-Flag tag antibody		Sigma	1:1000
15	Anti-His tag antibody		Proteintech	1:5000
16	Anti Myc tag antibody		Sigma	1:1000
17	Anti-HA tag antibody		Proteintech	1:1000
18	HRP conjugated Goat anti-Rabbit IgG		Thermo Scientific	1:5000
19	HRP conjugated Goat anti-Rabbit IgG		Thermo Scientific	1:5000
20	HRP conjugated Rabbit anti-goat IgG		Thermo Scientific	1:5000
21	HRP conjugated mouse anti-rabbit light chain specific		ECM Biosciences	1:5000
22	Phospho-Histone H2A.X (Ser139) Antibody		Cell Signaling Technology	1:1000
23	Histone H2A.X		Cell Signaling Technology	1:1000

List of primers

a) cDNA cloning primers

Name	Sequence (5’-3’)
EcoR1-FOXP1-F	CCGGAATTCATGATGCAAGAATCTGGGACTGAG
Not1-HIS-FOXP1-R	ATAAGAATGCGGCCGCTCAATGGTGATGGTGATGATG CTCCATGTCCTCGTTTACTGG
EcoR1-BIRC5-F	CCGGAATTCCCGGCCACCATGGGTGCCCCGACGTTGC CC
BamH1-BIRC5-R	CGCGGATCCGCGTCAATCCATGGCAGCCAGCTGCTCG
Xba1-AURKA-F	TGCTCTAGAGCAGCCACCATGGACCGATCTAAAGAAAA CTGC
BamH1-AURKA-R	CGCGGATCCGCGCTAAGACTGTTTGCTAGCTGATTC
EcoR1-FlagUB-F	CGGAATTCGCCACCATGGACTACAAGGACGACGATGA CAAGCAGATCTTCGTGAAGACC
Xho1-UB-R	CCGCTCGAGCGGTCAACCACCTCTCAGACGCAGGACC
Sma1-UBE1-F	TCCCCCGGGGATGAAGCGGCTCCAGACATCCAG
Not1-His-UBE1-R	ATAAGAATGCGGCCGCTCAATGGTGATGGTGATGATG GCGGATGGTGTATCGGACATAG
EcoR1-UBCH5B-F	CCGGAATTCCGGATGGCTCTGAAGAGAATCCAC
Xho1-His-UBCH5B-R	CCGCTCGAGCGGTCAATGGTGATGGTGATGATGCATC GCATACTTCTGAGTCC
EcoR1-UBCH5C-F	CCGGAATTCCGGATGGCGCTGAAACGGATTAATAAG
Xho1-His-UBCH5C-R	CCGCTCGAGCGGTCAATGGTGATGGTGATGATGCATG GCATACTTCTGAGTCCATTCC
EcoR1-UBCH7-F	CCGGAATTCCGGATGGCGGCCAGCAGGAGGCTG
Xho1-His-UBCH7-R	CCGCTCGAGCGGTCAATGGTGATGGTGATGATGGTCC ACAGGTCGCTTTTCCC
EcoR1-HIS-FBXL7-F	CCGGAATTCCGGATGCATCATCACCATCACCATGGCG CGAACAATGGCAAAC
Not1-FBXL7-R	ATAAGAATGCGGCCGCTCAGAAGAAAGCCGGGTTGGT G
BamH1-Skp1-F	CGCGGATCCGCCACCATGCCTTCAATTAAGTTGCAGAG
Xho1-Skp1-R	CCGCTCGAGCGAAGACAAAACTGTGTGCTACCTAC
Sma1-HA-Cul1-F	TCCCCCGGGGATGTACCCATACGATGTTCCAGATTACG CTTCGTCAACCCGGAGCCAGA
Not1-Cul1-R	ATAAGAATGCGGCCGCTTAAGCCAAGTAACTGTAGGTG
Kpn1-FBXW7-F	CGGGGTACCGAATTCGCCACCATGAATCAGGAACTGC

	TCTCTG
Kpn1-FBXW7-R	CGGGGTACCCTTCATGTCCACATCAAAGTCC
Not1-FBXO10-F	AGAATGCGGCCGCGAGGCTGGTGGCCTCCCCTTGG
Xba1-FBXO10-R	TGCTCTAGAGCATCACAGGATGGTGCAGAAGACAC
Not1-FBXO16-F	AGAATGCGGCCGCGCATTTGCACCTCCAAAAAACAC
Xba1-FBXO16-R	TGCTCTAGAGCATTAGGGACATAGTGGGAAGG
Not1-FBX1-F	AGAATGCGGCCGCGGGAGCGGCGGCGTGGTCC
Xba1-FBX1-R	TGCTCTAGAGCATTACAGCCTCACAAGGCCCAGG
Not1-FBXL7-F	AGAATGCGGCCGCGGCGCGAACAATGGCAAACAG
Xba1-FBXL7-R	TGCTCTAGAGCATCAGAAGAAAGCCGGGTTGGTG
Not1-FBXL13-F	AGAATGCGGCCGCACTCCGGAATTGATGATAAAAGC
Xba1-FBXL13-R	TGCTCTAGAGCATCACGCTGCTTGGTCTTCAC
Not1-FBXO10-F	AGAATGCGGCCGCGAGGCTGGTGGCCTCCCCTTGG
Xba1-FBXO10-R	TGCTCTAGAGCATCACAGGATGGTGCAGAAGACAC
Not1-FBXO16-F	AGAATGCGGCCGCGCATTTGCACCTCCAAAAAACAC
Xba1-FBXO16-R	TGCTCTAGAGCATTAGGGACATAGTGGGAAGG

Promoter cloning primers

**Name**	**Sequence (5'-3')**
Xho1-1.5kb-FBXL7-F	CCGCTCGAGACTAATACAGACCAGGTGAGCAG
Xho1-1k-FBXL7-F	CCGCTCGAGACGTAATTACAAAACGCCTGG
Xho1-500bp-FBXL7-F	CCGCTCGAGTCCTGGGACCGACTCCCTGCTAAG
Xho1-212bp-FBXL7-F	CCGCTCGAGGCTTCGCTCCGAAGGGTTCGCGCG
HindIII-FBXL7-R	CCCAAGCTTCCTGTAGCCGTCATGCCCGCC
HindIII-BIRC5P-F	CCCAAGCTTCCCGGTTAGCGAGCCAATCAGC
Nco1-BIRC5P-R	CATGCCATGGTTCTTGAATGTAGAGATGCGGTGG

Chip primers

Name	Sequence (5'-3')
FBXL7Chip1-F	CCCCAAGTTTTCCCTCCCTC
FBXL7Chip1-R	ATGAACTTCCAGGCGTCGTT
FBXL7Chip2-F	GCTCTGTGCTTGCCTCACTA
FBXL7Chip2-R	CCTTTGGTGTTCGCTGCATT
FBXL7Chip3-F	GGGACCCGCAACAAGTGG
FBXL7Chip3-R	ACAGCTGCAGCACTTACAAT

qPCR primers

Name	Sequence (5'-3')
AURKA-F	CCACCTTCGGCATCCTAATA
AURKA-R	TCCAAGTGGTGCATATTCCA
ACTB-F	TTGCCGACAGGATGCAGAAGGA
ACTB-R	AGGTGGACAGCGAGGCCAGGAT
GAPDH-F	GAAGGTGAAGGTCGGAGTC
GAPDH-R	GAAGATGGTGATGGGATTTC
BIRC5-F	AGAACTGGCCCTTCTTGGAGG
BIRC5-R	CTTTTTATGTTCCTCTATGGGGTC
FBXL7-F	GATCACACGCCCACTAAAGC
FBXL7-R	CCTTCCATTCTGAAATCCTGGG

si/sh oligonucleotides

Name	Sequence (5'-3')
si/shAurA	GCACCACTTGGAACAGTTTAT
SiSurvivin	GCATTCGTCCGGTTGCGCT
ShGFP	GCAAGCTGACCCTGAAGTTCAT
