# Supplementary material for: Aurora kinase A regulates Survivin stability through targeting FBXL7 in gastric cancer drug resistance and prognosis
Source: Oncogenesis. 2017 Feb 20;6(2):e298–. doi: 10.1038/oncsis.2016.80 (PMC5337621; doi:10.1038/oncsis.2016.80)
Supplement: Supplementary Table 5 [file oncsis201680x9.pdf]

Supplementary Table 5. MatInspector Analysis

Matrix Family	Detailed Family Information	Matrix	Detailed Matrix Information	Opt.	Strand	Core sim.	Matrix sim.	Mat. sim. - opt.	Sequence
V$FKHD	Fork head domain factors	V$FOXP1_ES.01	Alternative splicing variant of FOXP1, activated in ESCs	1	+	1	1	0	aatacaaAACAtttgat
V$FKHD	Fork head domain factors	V$FOXP1_ES.01	Alternative splicing variant of FOXP1, activated in ESCs	1	+	1	1	0	ggaataaAACAttaagg
V$TALE	TALE homeodomain class recognizing TG motifs	V$TGIF.01	TG-interacting factor belonging to TALE class of homeodomain factors	1	+	1	1	0	ctgaaaggtGTCAgaat
V$FKHD	Fork head domain factors	V$FOXP1_ES.01	Alternative splicing variant of FOXP1, activated in ESCs	1	+	1	1	0	acagtaaAACAgagcca
V$TAIP	TGF-beta induced apoptosis proteins	V$CSRNP1.01	Cysteine-serine-rich nuclear protein 1 (AXUD1, AXIN1 up-regulated 1)	1	-	1	1	0	AGAGtga
V$TALE	TALE homeodomain class recognizing TG motifs	V$TGIF.01	TG-interacting factor belonging to TALE class of homeodomain factors	1	-	1	1	0	taaatgtctGTCAaatt
V$FKHD	Fork head domain factors	V$FOXP1_ES.01	Alternative splicing variant of FOXP1, activated in ESCs	1	-	1	1	0	gggataaAACAtccata
V$FKHD	Fork head domain factors	V$FOXP1_ES.01	Alternative splicing variant of FOXP1, activated in ESCs	1	-	1	1	0	tgtttaaAACAttatag
V$ZF02	C2H2 zinc finger transcription factors 2	V$ZNF300.01	KRAB-containing zinc finger protein 300	0.99	+	1	1	0.01	ttttccgCCCCagtctccaaaca
V$BRAC	Brachyury gene, mesoderm developmental factor	V$TBX5.01	T-Box factor 5 site (TBX5), mutations related to Holt-Oram syndrome	0.99	+	1	1	0.01	acacctgaaaGGTGtcagaattagacacg
V$SMAD	Vertebrate SMAD family of transcription factors	V$SMAD3.01	Smad3 transcription factor involved in TGF-beta signaling	0.99	+	1	0.995	0.005	aaaGTCTggct
V$RUSH	SWI/SNF related nucleophosphoproteins with a RING finger DNA binding motif	V$SMARCA3.02	SWI/SNF related, matrix associated, actin dependent regulator of chromatin, subfamily a, member 3	0.98	+	1	0.986	0.006	atacACTTtta
V$MOKF	Mouse Krueppel like factor	V$MOK2.02	Ribonucleoprotein associated zinc finger protein MOK-2 (human)	0.98	-	1	0.994	0.014	aaggggcagcgcgCCTTttcg
V$RUSH	SWI/SNF related nucleophosphoproteins with a RING finger DNA binding motif	V$SMARCA3.02	SWI/SNF related, matrix associated, actin dependent regulator of chromatin, subfamily a, member 3	0.98	-	1	0.986	0.006	ccggACTTttg
V$SORY	SOX/SRY-sex/testis determinig and related HMG box factors	V$SOX6.01	SRY (sex determining region Y)-box 6	0.97	+	1	1	0.03	tttaaACAAaggataacaccaac
V$HOMF	Homeodomain transcription factors	V$MSX.01	Homeodomain proteins MSX-1 and MSX-2	0.97	+	1	0.994	0.024	tttaattTAATttgcaaca
V$CIZF	CAS interating zinc finger protein	V$NMP4.01	NMP4 (nuclear matrix protein 4) / CIZ (Cas-interacting zinc finger protein)	0.97	+	1	0.993	0.023	ggAAAAactgc
V$ZFXY	Zfx and Zfy - transcription factors implicated in mammalian sex determination	V$ZFX.01	X-linked zinc finger protein	0.97	+	1	0.993	0.023	ggGGCCtggag
V$CART	Cart-1 (cartilage homeoprotein 1)	V$S8.01	Binding site for S8 type homeodomains	0.97	+	1	0.992	0.022	atacgTAATtacaaaacgcct
V$HOMF	Homeodomain transcription factors	V$MSX.01	Homeodomain proteins MSX-1 and MSX-2	0.97	+	1	0.989	0.019	acactttTAATttaatttg
V$NKXH	NKX homeodomain factors	V$NKX25.05	Homeodomain factor Nkx-2.5/Csx	0.97	+	1	0.981	0.011	cacggTGAGtgccctggtc
V$SORY	SOX/SRY-sex/testis determinig and related HMG box factors	V$SOX6.01	SRY (sex determining region Y)-box 6	0.97	+	1	0.974	0.004	tcactACAAagaaatttgcacgg
V$HOMF	Homeodomain transcription factors	V$MSX.01	Homeodomain proteins MSX-1 and MSX-2	0.97	+	1	0.972	0.002	ttagataTAATttgacaga
V$CIZF	CAS interating zinc finger protein	V$NMP4.01	NMP4 (nuclear matrix protein 4) / CIZ (Cas-interacting zinc finger protein)	0.97	+	1	0.971	0.001	ggAAAAatcag
V$ZFHX	Two-handed zinc finger homeodomain transcription factors	V$AREB6.02	AREB6 (Atp1a1 regulatory element binding factor 6)	0.97	+	1	0.971	0.001	cataCACCtgaaa
V$CART	Cart-1 (cartilage homeoprotein 1)	V$S8.01	Binding site for S8 type homeodomains	0.97	-	1	0.992	0.022	ttttgTAATtacgtatttctt
V$HOMF	Homeodomain transcription factors	V$MSX.01	Homeodomain proteins MSX-1 and MSX-2	0.97	-	1	0.977	0.007	actatttTAATtcatcttt
V$HOMF	Homeodomain transcription factors	V$MSX.01	Homeodomain proteins MSX-1 and MSX-2	0.97	-	1	0.977	0.007	ccgtgtcTAATtctgacac
V$CEBP	Ccaat/Enhancer Binding Protein	V$CEBPE.02	CCAAT/enhancer binding protein (C/EBP), epsilon	0.97	-	0.96	0.975	0.005	cctcttttGGAAatt
V$E2FF	E2F-myc activator/cell cycle regulator	V$E2F4.01	E2F transcription factor 4, p107/p130-binding protein	0.96	+	1	0.986	0.026	ccgggGCGGggcaggga

V$YY1F	Activator/repressor binding to transcription initiation site	V$YY2.01	Transcription factor yin yang 2	0.96	+	1	0.973	0.013	aaagggCCATtttaaagctgtag
V$GFI1	Growth factor independence transcriptional repressor	V$GFI1.01	Growth factor independence 1 zinc finger protein acts as transcriptional repressor	0.96	+	1	0.973	0.013	aaaAATCacagaaac
V$YY1F	Activator/repressor binding to transcription initiation site	V$YY2.01	Transcription factor yin yang 2	0.96	+	1	0.972	0.012	tgtcttCCATtttccgccccagt
V$KLFS	Krueppel like transcription factors	V$GKLF.02	Gut-enriched Krueppel-like factor	0.96	+	1	0.961	0.001	aacaccAAAGggccatt
V$KLFS	Krueppel like transcription factors	V$GKLF.02	Gut-enriched Krueppel-like factor	0.96	+	1	0.961	0.001	ttaaacAAAGgataaca
V$KLFS	Krueppel like transcription factors	V$GKLF.02	Gut-enriched Krueppel-like factor	0.96	-	1	0.985	0.025	ccttttAAAGgggcagc
V$FKHD	Fork head domain factors	V$FREAC7.01	Fork head related activator-7 (FOXL1)	0.96	-	1	0.983	0.023	ttaaaaTAAAcacgcat
V$FKHD	Fork head domain factors	V$FREAC7.01	Fork head related activator-7 (FOXL1)	0.96	-	1	0.976	0.016	catacaTAAAtatgcgt
V$RBP2	Retinoblastoma-binding proteins with demethylase activity	V$PLU1_JARID1B.01	Jumonji, AT rich interactive domain 1B	0.96	-	1	0.975	0.015	GCACagagc
V$YY1F	Activator/repressor binding to transcription initiation site	V$YY2.01	Transcription factor yin yang 2	0.96	-	1	0.971	0.011	aaacatCCATatttcaagatttt
V$ETSF	Human and murine ETS1 factors	V$ETV1.02	Ets variant 1	0.96	-	1	0.968	0.008	cccggggaGGAAggccggccc
V$NFAT	Nuclear factor of activated T-cells	V$NFAT.01	Nuclear factor of activated T-cells	0.95	+	1	0.972	0.022	gacagtGGAAaaatcagca
V$WHNF	Winged helix binding sites	V$WHN.01	Winged helix protein, involved in hair keratinization and thymus epithelium differentiation	0.95	+	1	0.969	0.019	acgACGCctgg
V$GATA	GATA binding factors	V$GATA1.03	GATA-binding factor 1	0.95	+	1	0.966	0.016	tgaaGATAaagta
V$NFAT	Nuclear factor of activated T-cells	V$NFAT.01	Nuclear factor of activated T-cells	0.95	+	1	0.966	0.016	ctacaaGGAAaaactgctc
V$WHNF	Winged helix binding sites	V$WHN.01	Winged helix protein, involved in hair keratinization and thymus epithelium differentiation	0.95	+	1	0.966	0.016	ttgACGCtggg
V$HBOX	Homeobox transcription factors	V$GSH2.01	Homeodomain transcription factor Gsh-2	0.95	+	1	0.964	0.014	ttaattTAATttgcaacat
V$HOMF	Homeodomain transcription factors	V$HHEX.01	Hematopoietically expressed homeobox, proline-rich homeodomain protein	0.95	+	1	0.957	0.007	atatacactttTAATttaa
V$HOMF	Homeodomain transcription factors	V$BSX.01	Brain specific homeobox	0.95	+	1	0.954	0.004	aaatacgtAATTacaaaac
V$HEAT	Heat shock factors	V$HSF2.02	Heat shock factor 2	0.95	+	1	0.951	0.001	ttttaaagctgtAGAAgtgtcacat
V$HICF	Krueppel-like C2H2 zinc finger factors hypermethylated in cancer	V$HIC1.02	Hypermethylated in cancer 1 (secondary DNA binding preference)	0.95	+	1	0.951	0.001	cggTGCCcaggct
V$NKXH	NKX homeodomain factors	V$NKX31.04	NK3 homeobox 1	0.95	-	1	0.989	0.039	tattttaAGTGcgtgggca
V$AHRR	AHR-arnt heterodimers and AHR-related factors	V$AHRARNT.03	DRE (dioxin response elements), XRE (xenobiotic response elements) bound by AHR/ARNT heterodimers	0.95	-	1	0.973	0.023	tattttaagtGCGTgggcatttaaa
V$HOMF	Homeodomain transcription factors	V$HHEX.01	Hematopoietically expressed homeobox, proline-rich homeodomain protein	0.95	-	1	0.971	0.021	aattcatctttTAATatct
V$HOMF	Homeodomain transcription factors	V$HHEX.01	Hematopoietically expressed homeobox, proline-rich homeodomain protein	0.95	-	1	0.967	0.017	aaagactatttTAATtcat
V$AHRR	AHR-arnt heterodimers and AHR-related factors	V$AHRARNT.03	DRE (dioxin response elements), XRE (xenobiotic response elements) bound by AHR/ARNT heterodimers	0.95	-	1	0.96	0.01	tgcatatgatGCGTgaatacagaac
V$HOMF	Homeodomain transcription factors	V$BSX.01	Brain specific homeobox	0.95	-	1	0.954	0.004	cgttttgtAATTacgtatt
V$SMAD	Vertebrate SMAD family of transcription factors	V$GC_SBE.01	GC-rich Smad1/5 binding element	0.94	+	1	1	0.06	cggggCTCCag

V$SORY	SOX/SRY-sex/testis determinig and related HMG box factors	V$SOX3.01	SRY-box containing gene 3	0.94	+	1	0.985	0.045	ttgttaCAAAggaaaattgtagt
V$SORY	SOX/SRY-sex/testis determinig and related HMG box factors	V$SOX3.01	SRY-box containing gene 3	0.94	+	1	0.964	0.024	cgctaaCAAAagtccggcccgcg
V$CDXF	Vertebrate caudal related homeodomain protein	V$CDX1.01	Intestine specific homeodomain factor CDX-1	0.94	+	1	0.953	0.013	caagcctTTTAagaaatac
V$HAML	Human acute myelogenous leukemia factors	V$AML2.01	RUNX3 (Runt-related transcription factor 3), AML2 (Acute myeloid leukemia 2)	0.94	+	1	0.941	0.001	gcaTGTGgtcacaag
V$SORY	SOX/SRY-sex/testis determinig and related HMG box factors	V$SOX3.01	SRY-box containing gene 3	0.94	+	0.817	0.94	0	agtaaaTAAAggaaattacccaa
V$STAT	Signal transducer and activator of transcription	V$STAT3.02	Signal transducer and activator of transcription 3	0.94	+	1	0.94	0	acatTTCCtggctctgaag
V$SMAD	Vertebrate SMAD family of transcription factors	V$GC_SBE.01	GC-rich Smad1/5 binding element	0.94	-	1	1	0.06	tgcggCTCCag
V$BZIP	Heterodimers between bZIP family members	V$CEBPE_ATF4.01	Heterodimer of CEBP epsilon and ATF4	0.94	-	1	0.985	0.045	agtgagGCAAgca
V$HAML	Human acute myelogenous leukemia factors	V$AML2.01	RUNX3 (Runt-related transcription factor 3), AML2 (Acute myeloid leukemia 2)	0.94	-	1	0.978	0.038	tctTGTGgttggtgt
V$SATB	Special AT-rich sequence binding protein	V$SATB1.01	Special AT-rich sequence-binding protein 1, predominantly expressed in thymocytes, binds to matrix attachment regions (MARs)	0.94	-	1	0.968	0.028	aatAATAatctattt
V$CLOX	CLOX and CLOX homology (CDP) factors	V$CDP.02	Transcriptional repressor CDP	0.94	-	1	0.961	0.021	cactggcCAATaatatgtgcttc
V$CEBP	Ccaat/Enhancer Binding Protein	V$CEBPB.01	CCAAT/enhancer binding protein beta	0.94	-	0.94	0.952	0.012	ggcgttttGTAAtta
V$CDXF	Vertebrate caudal related homeodomain protein	V$CDX1.01	Intestine specific homeodomain factor CDX-1	0.94	-	1	0.949	0.009	ttcatctTTTAatatctag
V$CEBP	Ccaat/Enhancer Binding Protein	V$CEBPA.01	CCAAT/enhancer binding protein alpha	0.94	-	0.917	0.947	0.007	ctatttggGTAAttt
V$SPZ1	Testis-specific bHLH-Zip transcription factors	V$SPZ1.01	Spermatogenic Zip 1 transcription factor	0.94	-	1	0.946	0.006	gGGAGggaaaa
V$SORY	SOX/SRY-sex/testis determinig and related HMG box factors	V$SOX3.01	SRY-box containing gene 3	0.94	-	0.824	0.943	0.003	aacagaAAAAggaaattctcctt
V$IRFF	Interferon regulatory factors	V$IRF4.01	Interferon regulatory factor (IRF)-related protein (NF-EM5, PIP, LSIRF, ICSAT)	0.94	-	1	0.942	0.002	agatatcatgtactGAAAgaaaaaa
V$CLOX	CLOX and CLOX homology (CDP) factors	V$CDP.02	Transcriptional repressor CDP	0.94	-	1	0.94	0	ccagcgtCAATcacagctggagc
V$ETSF	Human and murine ETS1 factors	V$ERG.02	v-ets erythroblastosis virus E26 oncogene homolog	0.93	+	1	0.978	0.048	taaataaaGGAAattacccaa
V$BEDF	BED subclass of zinc-finger proteins	V$ZBED4.01	Zinc finger, BED-type containing 4; GC-box binding sites	0.93	+	1	0.955	0.025	gctGGGCggcgagag
V$ETSF	Human and murine ETS1 factors	V$ERG.02	v-ets erythroblastosis virus E26 oncogene homolog	0.93	+	1	0.949	0.019	gttacaaaGGAAaattgtagt

V$PBXC	PBX - MEIS complexes	V$PBX3.01	Pre-B-cell leukemia homeobox 3	0.93	+	1	0.945	0.015	gctgtgatTGACgctgg
V$BEDF	BED subclass of zinc-finger proteins	V$ZBED4.01	Zinc finger, BED-type containing 4; GC-box binding sites	0.93	+	1	0.942	0.012	ccgGGGCggggcagg
V$ETSF	Human and murine ETS1 factors	V$ERG.02	v-ets erythroblastosis virus E26 oncogene homolog	0.93	+	1	0.932	0.002	ctgagccaGGAAacctccttc
V$ETSF	Human and murine ETS1 factors	V$ERG.02	v-ets erythroblastosis virus E26 oncogene homolog	0.93	-	1	0.97	0.04	cagaaaaaGGAAattctcctt
V$ETSF	Human and murine ETS1 factors	V$ERG.02	v-ets erythroblastosis virus E26 oncogene homolog	0.93	-	1	0.953	0.023	cagagccaGGAAatgtatttt
V$KLFS	Krueppel like transcription factors	V$BTEB3.01	Basic transcription element (BTE) binding protein, BTEB3, FKLF-2	0.93	-	1	0.948	0.018	tagcagGGAGtcggtcc
V$HAND	Twist subfamily of class B bHLH transcription factors	V$TH1E47.01	Thing1/E47 heterodimer, TH1 bHLH member specific expression in a variety of embryonic tissues	0.93	-	1	0.934	0.004	ccaggagagCCAGactttccc
V$ARID	AT rich interactive domain factor	V$BRIGHT.01	Bright, B cell regulator of IgH transcription	0.92	+	1	0.97	0.05	gatgaATTAaaatagtctttc
V$DLXF	Distal-less homeodomain transcription factors	V$DLX2.01	Distal-less homeobox 2	0.92	+	1	0.97	0.05	agaaatacgtAATTacaaa
V$KLFS	Krueppel like transcription factors	V$KLF7.01	Kruppel-like factor 7 (ubiquitous, UKLF)	0.92	+	1	0.961	0.041	ccggGGCGgggcaggga
V$SORY	SOX/SRY-sex/testis determinig and related HMG box factors	V$HMGIY.01	HMGI(Y) high-mobility-group protein I (Y), architectural transcription factor organizing the framework of a nuclear protein-DNA transcriptional complex	0.92	+	1	0.95	0.03	aggaAATTacccaaatagaatct
V$BRNF	Brn POU domain factors	V$BRN2.03	Brn-2, POU-III protein class	0.92	+	1	0.949	0.029	acacttttaATTTaatttg
V$PIT1	GHF-1 pituitary specific pou domain transcription factor	V$PIT1.01	Pit1, GHF-1 pituitary specific pou domain transcription factor	0.92	+	0.839	0.948	0.028	tgtatGTATgcatca
V$STAT	Signal transducer and activator of transcription	V$STAT5B.01	Signal transducer and activator of transcription 5B	0.92	+	1	0.94	0.02	aaaaatcacAGAAacgagg
V$HOMF	Homeodomain transcription factors	V$HMX3.02	Hmx3/Nkx5-1 homeodomain transcription factor	0.92	+	1	0.936	0.016	cgctgcccctTTAAaggc
V$SORY	SOX/SRY-sex/testis determinig and related HMG box factors	V$HMGIY.01	HMGI(Y) high-mobility-group protein I (Y), architectural transcription factor organizing the framework of a nuclear protein-DNA transcriptional complex	0.92	+	1	0.933	0.013	cataAATTccaaaagaggctat
V$SORY	SOX/SRY-sex/testis determinig and related HMG box factors	V$HMGIY.01	HMGI(Y) high-mobility-group protein I (Y), architectural transcription factor organizing the framework of a nuclear protein-DNA transcriptional complex	0.92	+	1	0.932	0.012	aagaAATTgcacggtgagtgcc
V$HOMF	Homeodomain transcription factors	V$HMX3.02	Hmx3/Nkx5-1 homeodomain transcription factor	0.92	+	1	0.932	0.012	gcccacgcacTTAAaatag
V$AP1R	MAF and AP1 related factors	V$MAFA.01	Lens-specific Maf/MafA-sites	0.92	+	1	0.928	0.008	agtggaaaaatcAGCAcaataca
V$MYT1	MYT1 C2HC zinc finger protein	V$MYT1L.01	Myelin transcription factor 1-like, neuronal C2HC zinc finger factor 1	0.92	+	0.818	0.927	0.007	tgaaAGGTgtcag
V$PIT1	GHF-1 pituitary specific pou domain transcription factor	V$PIT1.01	Pit1, GHF-1 pituitary specific pou domain transcription factor	0.92	+	1	0.925	0.005	catatTTATgtatgt
V$ABDB	Abdominal-B type homeodomain transcription factors	V$HOXD10.01	Homeobox D10	0.92	+	1	0.924	0.004	aaagggaaTAAAacatt
V$BTBF	BTB/POZ (broad complex, TramTrack, Bric-a-brac/pox viruses and zinc fingers) transcription factor	V$KAISO.01	Transcription factor Kaiso, ZBTB33	0.92	+	1	0.922	0.002	ctccCTGCtaa

V$ETSF	Human and murine ETS1 factors	V$CETS1P54.01	c-Ets-1(p54)	0.92	+	0.843	0.922	0.002	cgacgcCTGGaagttcatctt
V$SORY	SOX/SRY-sex/testis determinig and related HMG box factors	V$HMGIY.01	HMGI(Y) high-mobility-group protein I (Y), architectural transcription factor organizing the framework of a nuclear protein-DNA transcriptional complex	0.92	+	1	0.921	0.001	ggagAATTtcctttttctgttta
V$PAX5	PAX-2/5/8 binding sites	V$PAX2.02	Paired box protein 2	0.92	-	0.962	0.97	0.05	gcatatgatgcgTGAAtacagaaccaggc
V$PIT1	GHF-1 pituitary specific pou domain transcription factor	V$PIT1.01	Pit1, GHF-1 pituitary specific pou domain transcription factor	0.92	-	0.855	0.954	0.034	tagatATATgcatat
V$CAAT	CCAAT binding factors	V$NFY.04	Nuclear factor Y (Y-box binding factor)	0.92	-	1	0.953	0.033	ctggCCAAtaatatg
V$SORY	SOX/SRY-sex/testis determinig and related HMG box factors	V$HMGIY.01	HMGI(Y) high-mobility-group protein I (Y), architectural transcription factor organizing the framework of a nuclear protein-DNA transcriptional complex	0.92	-	1	0.95	0.03	tggaAATTtatgtgacacttcta
V$ARID	AT rich interactive domain factor	V$BRIGHT.01	Bright, B cell regulator of IgH transcription	0.92	-	1	0.949	0.029	tgcaaATTAaattaaaagtgt
V$ARID	AT rich interactive domain factor	V$BRIGHT.01	Bright, B cell regulator of IgH transcription	0.92	-	1	0.948	0.028	attaaATTAaaagtgtatatt
V$SORY	SOX/SRY-sex/testis determinig and related HMG box factors	V$HMGIY.01	HMGI(Y) high-mobility-group protein I (Y), architectural transcription factor organizing the framework of a nuclear protein-DNA transcriptional complex	0.92	-	1	0.946	0.026	aggaAATTctccttaatgtttta
V$HOMF	Homeodomain transcription factors	V$HMX3.02	Hmx3/Nkx5-1 homeodomain transcription factor	0.92	-	1	0.936	0.016	gcgcggcctttTTAAagggg
V$NACA	Nascent polypeptide associated complex and coactivator alpha	V$NACA1.01	Nascent polypeptide-associated complex subunit alpha 1	0.92	-	1	0.934	0.014	agcaCAGAgcagt
V$SORY	SOX/SRY-sex/testis determinig and related HMG box factors	V$SOX2.01	SRY-box containing gene 2	0.92	-	1	0.929	0.009	ttgtaACAAcgggataaaacatc
V$CEBP	Ccaat/Enhancer Binding Protein	V$CEBP.02	CCAAT/enhancer binding protein	0.92	-	1	0.929	0.009	gtagtgagGCAAgca
V$HOMF	Homeodomain transcription factors	V$HMX3.02	Hmx3/Nkx5-1 homeodomain transcription factor	0.92	-	1	0.922	0.002	acagcctattTTAAgtgcg
V$AP4R	AP4 and related proteins	V$AP4.02	Activator protein 4	0.92	-	1	0.921	0.001	aatcacAGCTggagccc
V$HESF	Vertebrate homologues of enhancer of split complex	V$HES1.01	Drosophila hairy and enhancer of split homologue 1 (HES-1)	0.92	-	0.944	0.921	0.001	gcgccagGCGCctcc
V$GATA	GATA binding factors	V$GATA1.04	GATA-binding factor 1	0.91	+	1	0.951	0.041	aaagGATAacacc
V$HOXC	HOX - PBX complexes	V$HOXC9.02	Member of the vertebrate HOX - cluster of homeobox factors	0.91	+	1	0.944	0.034	gattggaTTTAtgcttt
V$ZF07	C2H2 zinc finger transcription factors 7	V$ZNF263.02	Zinc finger protein 263, ZKSCAN12 (zinc finger protein with KRAB and SCAN domains 12)	0.91	+	1	0.936	0.026	gccttcCTCCccggg
V$BHLH	bHLH transcription factors expressed in muscle, intestine and stomach	V$MESP1_2.01	Mesoderm posterior 1 and 2	0.91	+	1	0.932	0.022	gcatCATAtgcat
V$GATA	GATA binding factors	V$GATA3.02	GATA-binding factor 3	0.91	+	1	0.929	0.019	tctAGATattaaa
V$DLXF	Distal-less homeodomain transcription factors	V$DLX5.01	Distal-less homeobox 5	0.91	+	1	0.922	0.012	aaataaaggaAATTaccca
V$NKX6	NK6 homeobox transcription factors	V$NKX61.01	NK6 homeobox 1	0.91	+	1	0.92	0.01	acttTTAAtttaatt
V$NKX6	NK6 homeobox transcription factors	V$NKX61.01	NK6 homeobox 1	0.91	+	1	0.92	0.01	taatTTAAtttgcaa
V$GATA	GATA binding factors	V$GATA3.02	GATA-binding factor 3	0.91	+	1	0.917	0.007	tttAGATataatt
V$SP1F	GC-Box factors SP1/GC	V$SP1.03	Stimulating protein 1, ubiquitous zinc finger transcription factor	0.91	+	1	0.914	0.004	acgctGGGCggcgagag
V$PAX6	PAX-4/PAX-6 paired domain binding sites	V$PAX4_PD.01	PAX4 paired domain binding site	0.91	+	0.793	0.913	0.003	tagACACggatgcgtgttt
V$SP1F	GC-Box factors SP1/GC	V$SP1.03	Stimulating protein 1, ubiquitous zinc finger transcription factor	0.91	+	1	0.912	0.002	gggcgGGGCagggaaag

V$DLXF	Distal-less homeodomain transcription factors	V$DLX1.01	DLX-1, -2, and -5 binding sites	0.91	-	1	0.976	0.066	ggcgttttgtAATTacgta
V$LHXF	Lim homeodomain factors	V$LMX1B.01	LIM-homeodomain transcription factor	0.91	-	1	0.963	0.053	tgaaagaaaaaaaTAATaatcta
V$ABDB	Abdominal-B type homeodomain transcription factors	V$HOXC13.01	Homeodomain transcription factor HOXC13	0.91	-	1	0.954	0.044	acatcctaaTAAAgacc
V$ZICF	Members of ZIC-family, zinc finger protein of the cerebellum	V$ZIC3.03	Zinc finger protein of the cerebellum (Zic3)	0.91	-	1	0.934	0.024	aatcaCAGCtggagc
V$GATA	GATA binding factors	V$GATA3.01	GATA-binding factor 3	0.91	-	1	0.932	0.022	acggGATAaaaca
V$BHLH	bHLH transcription factors expressed in muscle, intestine and stomach	V$MESP1_2.01	Mesoderm posterior 1 and 2	0.91	-	1	0.927	0.017	tatgCATAtgatg
V$FKHD	Fork head domain factors	V$HFH2.01	HNF-3/Fkh Homolog 2 (FOXD3)	0.91	-	0.892	0.922	0.012	aagaaaaaAATAataat
V$NKX6	NK6 homeobox transcription factors	V$NKX61.01	NK6 homeobox 1	0.91	-	1	0.917	0.007	tcttTTAAtatctag
V$EBOX	E-box binding factors	V$MYCMAX.03	MYC-MAX binding sites	0.91	-	0.789	0.917	0.007	agcgccaGGCGcctcct
V$GATA	GATA binding factors	V$GATA3.02	GATA-binding factor 3	0.91	-	1	0.916	0.006	aagAGATgaaaga
V$NKX6	NK6 homeobox transcription factors	V$NKX61.01	NK6 homeobox 1	0.91	-	1	0.914	0.004	tattTTAAttcatct
V$SP1F	GC-Box factors SP1/GC	V$SP1.03	Stimulating protein 1, ubiquitous zinc finger transcription factor	0.91	-	1	0.914	0.004	gactgGGGCggaaaatg
V$DLXF	Distal-less homeodomain transcription factors	V$DLX5.01	Distal-less homeobox 5	0.91	-	1	0.911	0.001	ctatttgggtAATTtcctt
V$GATA	GATA binding factors	V$GATA3.02	GATA-binding factor 3	0.91	-	1	0.91	0	tgtAGATgttaga
V$EVI1	EVI1-myeloid transforming protein	V$EVI1.07	Evi-1 zinc finger protein, carboxy-terminal zinc finger domain	0.9	+	1	0.961	0.061	attaaAAGAtgaattaa
V$HMTB	Human muscle-specific Mt binding site	V$MTBF.01	Muscle-specific Mt binding site	0.9	+	1	0.959	0.059	aggtATTTa
O$VTBP	Vertebrate TATA binding protein factor	O$VTATA.01	Cellular and viral TATA box elements	0.9	+	1	0.953	0.053	tgttaTAAAattctagg
V$MYOD	Myoblast determining factors	V$MYOGENIN.02	Myogenic bHLH protein myogenin (myf4)	0.9	+	1	0.943	0.043	gggctcCAGCtgtgatt
V$XBBF	X-box binding factors	V$XBOX.01	Motif bound by regulatory factor X (RFX) proteins	0.9	+	0.875	0.916	0.016	tcctccccgGGGCtccagc
V$BRNF	Brn POU domain factors	V$TST1.01	POU-factor Tst-1/Oct-6	0.9	+	1	0.902	0.002	gatgaATTAaaatagtctt
V$MYBL	Cellular and viral myb-like transcriptional regulators	V$VMYB.05	v-Myb, variant of AMV v-myb	0.9	-	1	0.964	0.064	cctttgtaacAACGggataaa
V$MYOD	Myoblast determining factors	V$MYOGENIN.03	Myogenic bHLH protein myogenin (myf4)	0.9	-	1	0.962	0.062	caatcaCAGCtggagcc
V$GATA	GATA binding factors	V$GATA2.02	GATA-binding factor 2	0.9	-	1	0.954	0.054	tagaGATAtcaaa
V$EVI1	EVI1-myeloid transforming protein	V$EVI1.07	Evi-1 zinc finger protein, carboxy-terminal zinc finger domain	0.9	-	1	0.948	0.048	tctatAAGAtgaacttc
V$GFI1	Growth factor independence transcriptional repressor	V$GFI1.02	Growth factor independence 1	0.9	-	1	0.942	0.042	gtcAATCacagctgg
V$FKHD	Fork head domain factors	V$HNF3B.04	HNF-3-beta, Forkhead box protein A2 (FOXA2)	0.9	-	0.873	0.923	0.023	ttcatgtaAATAcctag
V$BRNF	Brn POU domain factors	V$TST1.01	POU-factor Tst-1/Oct-6	0.9	-	1	0.92	0.02	gctgcATTAaacagaaaaa
V$AP2F	Activator protein 2	V$AP2.01	Activator protein 2	0.9	-	0.831	0.919	0.019	ctcGCCCgcgggccg
V$GATA	GATA binding factors	V$GATA2.02	GATA-binding factor 2	0.9	-	1	0.908	0.008	tctaGATAtcatg
O$PTBP	Plant TATA binding protein factor	O$PTATA.02	Plant TATA box	0.9	-	1	0.903	0.003	gagaTATAcatacat
