# Supplementary material for: Aurora kinase A regulates Survivin stability through targeting FBXL7 in gastric cancer drug resistance and prognosis
Source: Oncogenesis. 2017 Feb 20;6(2):e298–. doi: 10.1038/oncsis.2016.80 (PMC5337621; doi:10.1038/oncsis.2016.80)
Supplement: Supplementary Table 6 [file oncsis201680x10.pdf]

Supplementary Table 6

AURKA interacting nuclear transcription factors

S#	Accession#	Description
1	300300	CCAAT displacement protein, CDP [human, Peptide, 1505 aa]
2	112491340	Chain A, Nmr Structure Of The First Qrrm Domain Of Human Hnrnp F
3	119604960	Cofactor required for Sp1 transcriptional activation, subunit 7, 70kDa [Homo sapiens]
4	119615830	Forkhead box F1 [Homo sapiens]
5	119585901	forkhead box P1, isoform CRA_f [Homo sapiens]
6	119608416	General transcription factor IIIC, polypeptide 4, 90kDa, isoform CRA_a [Homo sapiens]
7	119583084	heterogeneous nuclear ribonucleoprotein K, isoform CRA_f [Homo sapiens]
8	17975763	myelin transcription factor 1 [Homo sapiens]
9	119613450	nuclear transcription factor, X-box binding-like 1, isoform CRA_d [Homo sapiens]
10	115529451	nucleolar transcription factor 1 isoform b [Homo sapiens]
11	119582250	POU domain, class 4, transcription factor 3, isoform CRA_a [Homo sapiens]
12	730840	FACT complex subunit SSRP1; AltName: Full=Chromatin-specific transcription elongation factor 80 kDa subunit
13	118572657	RecName: Full=Gamma-interferon-inducible protein 16; Short=Ifi-16; AltName: Full=Interferon-inducible myeloid differentiation transcriptional activator
14	327478568	RecName: Full=Myelin transcription factor 1-like protein; Short=MyT1-L; Short=MyT1L
15	189029591	RecName: Full=NK1 transcription factor-related protein 1; AltName: Full=Homeobox protein 153; Short=HPX-153; AltName: Full=Homeobox protein SAX-2; AltName: Full=NKX-1.1
16	3914276	RecName: Full=Paired box protein Pax-4
17	71152211	RecName: Full=Protein AATF; AltName: Full=Apoptosis-antagonizing transcription factor; AltName: Full=Rb-binding protein Che-1
18	296452989	RecName: Full=RE1-silencing transcription factor; AltName: Full=Neural-restrictive silencer factor; AltName: Full=X2 box repressor
19	1711552	RecName: Full=Signal transducer and activator of transcription 2; AltName: Full=p113
20	51338743	RecName: Full=T-box transcription factor TBX10; Short=T-box protein 10
21	12644232	RecName: Full=Transcription factor SOX-18
22	74745081	RecName: Full=Transcriptional protein SWT1
23	311033500	Transcriptional regulator ATRX; AltName: Full=ATP-dependent helicase ATRX
24	3915889	Transcriptional repressor protein YY1;Full=Yin and yang 1; Short=YY-1
25	108935822	Zinc finger homeobox protein 3; Short=ZFH-3
26	119604970	SIN3 homolog B, transcription regulator (yeast), isoform CRA_a [Homo sapiens]
27	119580874	sterol regulatory element binding transcription factor 2, isoform CRA_b [Homo sapiens]
28	17933774	transcription elongation factor A protein-like 2 [Homo sapiens]
29	84627476	Transcription elongation regulator 1 [Homo sapiens]
30	119624760	transcription factor AP-2 beta (activating enhancer binding protein 2 beta), isoform CRA_a [Homo sapiens]
31	30581117	transcription factor SOX-30 isoform a [Homo sapiens]
32	98961135	transcription factor SPT20 homolog isoform a [Homo sapiens]
33	392933927	transcriptional repressor CTCFL isoform 3 [Homo sapiens]
34	119597100	transformation/transcription domain-associated protein, isoform CRA_b [Homo sapiens]
35	120538359	Zinc finger with KRAB and SCAN domains 2 [Homo sapiens]
